# Optimizing the growth and flowering of *Rosa hybrida* L. (Roses) through synergistic light and biostimulant management

**DOI:** 10.1186/s12870-026-09034-3

**Published:** 2026-05-26

**Authors:** Aly H. M. El-Naggar, Rania EL-Tanbouly, Gabriel F. M. Imhmd, Naglaa M. Esmaiel

**Affiliations:** 1 Department of Floriculture, Ornamental Horticulture and Landscape Design, Faculty of Agriculture (El-Shatby), Alexandria University, Alexandria, Egypt; 2 Faculty of Agriculture, Omar-AlMukhtar University, Al-Bayda, Libya; 3 Agricultural Research Centre, Horticulture Research Institute, Sabahia, Egypt

**Keywords:** Biofertilizer, Light intensity, Seaweed extract (SWE), Active dry yeast (ADY), Horticultural productivity, Flower quality

## Abstract

**Background:**

Optimizing the growth and flowering of ornamental Roses is critical for improving horticultural productivity. This study investigated the synergistic effects of light intensity and two biostimulants, seaweed extract (SWE) and active dry yeast (ADY), on the growth, flower quality, chlorophyll, and essential nutrients content of rose plants. A factorial experiment tested three light regimes (full sun, 65% and 75% shading) combined with two concentrations of SWE (1000 and 2000 mg·L⁻¹) and two concentrations of ADY (3000 and 4000 mg·L⁻¹).

**Results:**

Cultivation under shading (65% and 75%) consistently reduced vegetative growth and key nutrients elements, including the chlorophyll content (SPAD) and leaf concentrations of N, P, Zn, and Cu. The full-sun and 65% light conditions yielded the highest chlorophyll content, which were significantly greater than those of the 75% shade treatment. Nutrient analysis revealed that full sun maximized N, P, Zn, and Cu, whereas K was highest under 75% shade. Conversely, plants grown under full sun, when supplemented with biostimulants, presented significant and synergistic increases in growth, flower quality, and nutrient accumulation. The most effective treatment was the combination of full sun and 4000 mg·L⁻¹. This combination significantly optimized key horticultural traits and resulted in the highest N, Zn, and Cu levels.

**Conclusion:**

Overall, the findings indicate that the positive effects of biostimulants on roses depend strongly on light availability, supporting a sustainable strategy that integrates light management and biostimulants application to enhance commercial rose production.

**Supplementary Information:**

The online version contains supplementary material available at 10.1186/s12870-026-09034-3.

## Introduction

The genus Rosa, a member of the Rosaceae family, includes more than 200 species and numerous hybrids, making it one of the most economically important ornamental plants worldwide [1]. When cultivated across diverse climates, roses are prized for their aesthetic appeal and play a crucial role in the floriculture industry, which has expanded from domestic to international markets [2]. The consistent demand for high-quality rose production necessitates continuous research into methods that enhance both quantitative and qualitative traits, including optimal growth, flowering performance, and resilience to environmental stressors [3].

In horticulture, biostimulants have gained importance as sustainable alternatives to conventional agrochemicals for improving plant growth and stress tolerance. Among these, seaweed extracts (SWE) and active dry yeast (ADY) are particularly noteworthy. SWE are rich in bioactive compounds such as plant growth regulators (cytokinins, auxins, and gibberellins), polysaccharides (laminarin, alginate, and fucoidan), amino acids, betaines, and various micronutrients [4]. These components act synergistically to promote plant growth, increase nutrient uptake, and strengthen plant defenses against abiotic stresses such as drought and salinity by inducing antioxidant mechanisms [5–7]. Recent studies have further highlighted that SWE could improve rooting in rose cuttings, demonstrating its potential as a substitute for synthetic hormones in propagation protocols [8]. Furthermore, the combined application of SWE with other innovative technologies, such as oxygen nanobubbles and nanosilicon, has been shown to improve the morphological characteristics, quality, and vase life of cut rose flowers [9].

ADY, which is mainly composed of *Saccharomyces cerevisiae*, is another effective biostimulants that provides rich biochemical profiles, including proteins, amino acids, B-complex vitamins, enzymes, and natural phytohormones. These components influence plant development when applied as foliar sprays or soil amendments [10, 11]. Research indicates that ADY can increase chlorophyll content, nutrient uptake, and photosynthetic efficiency, largely through the production of plant growth regulators during yeast metabolism [12]. The application of ADY has been demonstrated to positively impact growth parameters, nutrient content (N, P, K), and flower production in various rose cultivars [13].

In addition to nutritional and biostimulants interventions, environmental factors, particularly light intensity, are critical determinants of rose growth and flowering. Roses are known to have high light requirements, and optimizing the light environment is crucial for maximizing yield and quality in commercial production [14]. Insufficient light intensity, especially during the winter months, can lead to reduced growth, poor flower quality, and lower yields [15]. Conversely, appropriate supplemental light spectra, particularly combinations of red and blue light, have been shown to improve growth, carbohydrate levels, photosynthetic capacity, and overall yield while also accelerating flowering in cut roses [15].

Despite extensive work on the individual effects of biostimulants and light management in different plant species [16–19], their combined, potentially synergistic interaction in rose cultivation remains poorly characterized, particularly under suboptimal light conditions. This gap in knowledge regarding how SWE and ADY interact with different light intensities to modulate vegetative growth, flower quality, chlorophyll, and essential nutrients content provides a key rationale for the present study.

In this context, the central hypothesis is that the combined application of biostimulants (SWE and ADY) with adjusted light regimes may produce more than additive effects, leading to improved growth and flower quality compared with either factor applied alone, and that under reduced light (shade-like) conditions biostimulants application may help to alleviate shade-induced stress by supporting photosynthetic capacity, nutrient status, and antioxidant defenses. To explore this hypothesis, the experiment compares multiple light intensities and spectral qualities (Full sun, 65%, and 75% shade), with and without SWE (1000 and 2000 mg L ^-1^) and ADY (3000 and 4000 mg L ^-1^), in order to assess whether observed responses are consistent with additive or synergistic interactions and to evaluate the extent to which biostimulants can mitigate the negative effects of low light on *Rosa hybrida* L. plants while informing the development of environmentally friendly cultivation strategies.

## Materials and methods

### Plant material and growth conditions

**Fig. 1:**
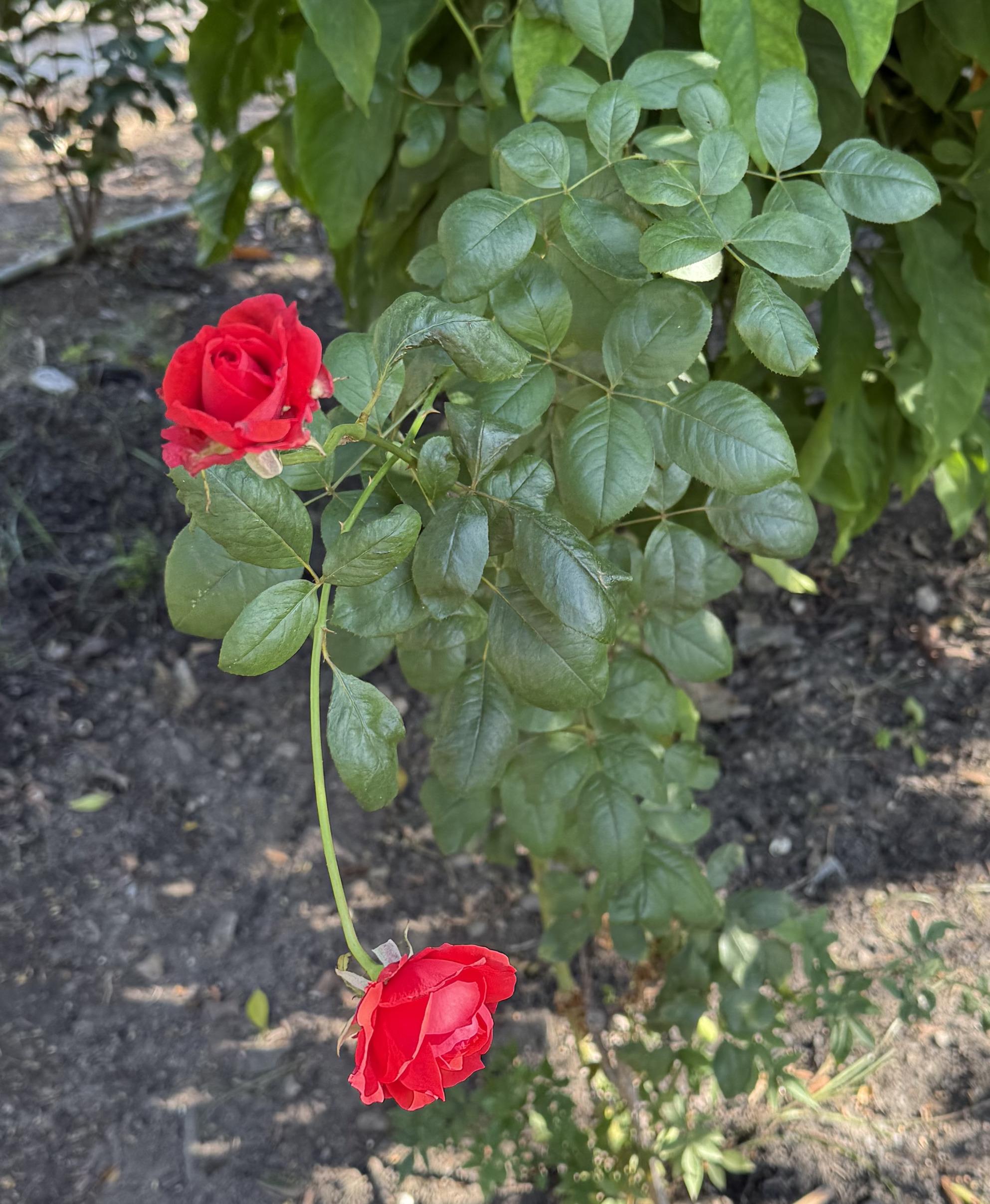
A representative image of *Rosa hybrida* L. cv. ‘Red Success’ (Locally named ‘Santrix’) plants used in the study, which were sourced from a commercial nursery “Al Masa” located at Borg El Arab, Alexandria Governorate, Egypt and grown under nursery conditions in Alexandria, Egypt. (Photo taken by Dr. Rania El-Tanbouly)

This study complied with relevant institutional, national, and international guidelines and legislation. The research did not involve human participants or animals, and no specific ethical approval was required. Plant material (*Rosa hybrids* L. cv. ‘Red Success’; Locally named ‘Santrix’) was used in accordance with applicable regulations (Fig. 1).

The experimental work was conducted under nursery conditions in Alexandria, Egypt. Uniform one-year-old *R. hybrida* plants were obtained from a commercial nursery “Al Masa” located at Borg El Arab, Alexandria Governorate, Egypt .The plant were initially identified by two of the authors based on morphological characteristics, and plant identity was then determined according to [20]. The cultivar determination was subsequently verified by Professor Dr. Mohamed Gamal Eltorky, Emerald Professor of Ornamental Plant Breeding at Alexandria University, Egypt. Following purchase, the plants were maintained in the nursery of the Department of Floriculture, Faculty of Agriculture, Alexandria University, Egypt, where the experiment was conducted.

The plants were transplanted into plastic pots (30 cm diameter), with one plant per pot, containing a 2:1 (v/v) mixture of peatmoss (pH 6.6, EC 0.6 dS m⁻¹) and perlite. Transplanting was carried out on February 28, 2023, and February 25, 2024, representing two consecutive experimental seasons. For each plant, three main branches were selected and pruned to a uniform length of 40 cm.

### Experimental design and treatments

**Fig. 2:**
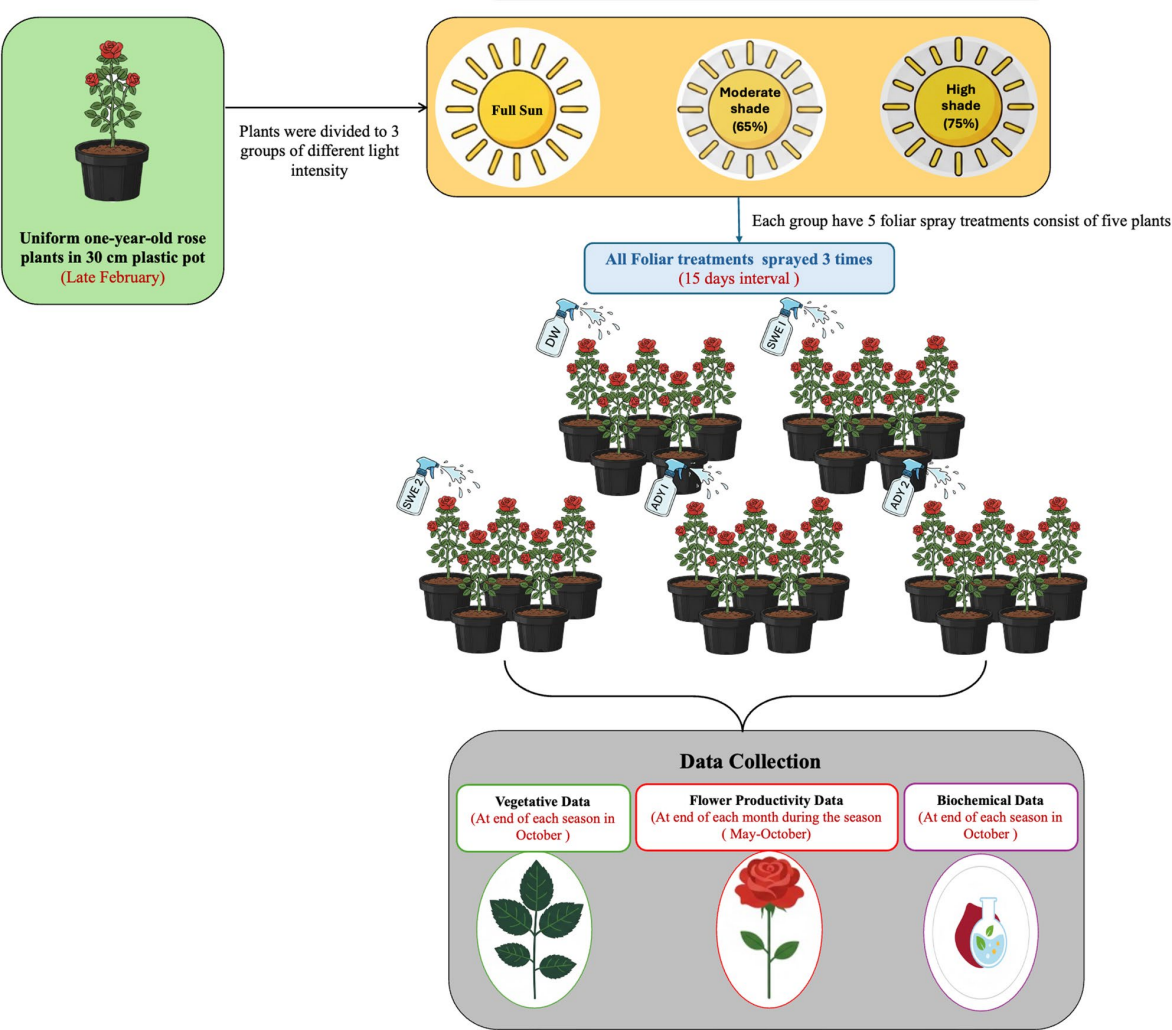
A schematic illustration of the experimental design showing experiment timeline. The factorial experimental layout included three light intensity regime (full sun, moderate shade, and high shade) and five foliar spray treatments: distilled water (DW), seaweed extract (SWE), and active yeast extract (ADY). All timelines are indicated in red coloured text

The experiment was conducted using a randomized complete block design (RCBD) with a 3 × 5 factorial arrangement of treatments. The two fixed factors were the light regime, which had three distinct levels, and the foliar spray treatment, which had five levels. Each of the five foliar spray treatments was applied to plants within each of the three light regimes, resulting in 15 treatment combinations. The experiment was replicated across three blocks. For each treatment combination within a block, a plot consisting of five individual plants was established, with each plant serving as an experimental unit (Fig. 2).

#### Light intensity conditions

The plants were divided into three light regimes (full sun, moderate shade, and high shade). Light intensity was monitored as photosynthetic photon flux density (PPFD, µmol m⁻² s⁻¹) using a digital illuminance meter (LI-250 A Light Meter, available at https://a.co/d/0goFzjbW), with measurements taken on clear days between 10:00 and 12:00 h at canopy height, at monthly intervals throughout the growing season. Across the experimental period, the full sun treatment averaged approximately 185–222 µmol m⁻² s⁻¹ for full sun (10,000–12,000 lx), 111–130 µmol m⁻² s⁻¹ for moderate shade under 65% Seran cover (6,000–7,000 lx), and 74–93 µmol m⁻² s⁻¹ for high shade under 75% Seran cover (4,000–5,000 lux).

#### Foliar application of seaweed extract and active dry yeast

Foliar treatments included seaweed extract (SWE) and active dry yeast (ADY). A powdered form of *Ascophyllum nodosum* (Acadian), obtained from Chema Industries Company, Egypt, was used as the SWE. The chemical composition of the Acadian extract powder is detailed in Table 1. SWE treatments were applied at concentrations of 1000 and 2000 mg L^− 1^. For ADY, *Saccharomyces cerevisiae* treatments were applied at concentrations of 3000 and 4000 mg. L^− 1^. The yeast suspension was prepared by dissolving ADY and sugar (1:1 w/w) in warm water (35–37 °C) and incubating at room temperature for 12 h prior to spraying to increase yeast activity, as described by Shalaby and El-Nady [21].

**Table 1:** Chemical composition of Acadian marine plant extract powder from *Ascophyllum nodosum*

Guaranteed minimum analysis		Physical properties	
T. Nitrogen (N)	0.7%	NPK and Minerals (ash)	45% − 55%
Av- Phosphoric Acid (P_2_O_5_)	0.2%	Moisture	Max 6.5%
Soluble Potash (K_2_O)	17%	Alginic Acid	Min 10%
		Mannitol	Min 4%
		Amino Acids	Min 4%
		Other organic matter derived from seaweed	Min 20%

Foliar spraying of SWE (1000 and 2000 mg L^− 1^) and ADY (3000 and 4000 mg L^− 1^) commenced one month after transplanting. The plants were sprayed to runoff three times at 15-day intervals in the early morning under windless conditions to avoid leaf burn and minimize evaporative loss, phytotoxicity risk, and potential spray drift between adjacent treatments. Distilled water served as the control treatment.

### Morphological measurements

To assess the efficacy and consistency of the treatments, the morphological characteristics of *R. hybrid*a plants were recorded over two successive growing seasons (2023 and 2024). This experimental design facilitated the evaluation of treatment responses under variable climatic conditions. Measurements were taken at the end of each season including the number of leaves, leaf area (cm² per plant), and leaf dry weight (g). While the flower productivity data including number of flowers per plant, flower fresh weight (g), stalk length (cm), and stalk thickness (mm) were collected at the end of each month (May- October) over two seasons. The leaf area was estimated from 20 uniformly sampled leaf discs per plant. The relationship between the fresh weight of these discs and their area was calculated, and the leaf area was determined via the following equation: El Naggar and Priya et al. [22, 23].

$$\text{Leaf area} \left(\mathrm{cm}^{2}\right)=\frac{\mathrm{x}}{\mathrm{Y}}\times\mathrm{M}$$

where x and Y represent the area (cm^2^) and weight (g) of the leaf segment, respectively, and where M represents the total leaf weight (g).

### Chlorophyll and nutrients content analysis

The analyses included relative chlorophyll content (SPAD units), macronutrients (%), and two essential micronutrients (Zn and Cu). Measuring nitrogen (N), phosphorus (P), potassium (K), zinc (Zn), and copper (Cu) in studies of roses and other plants is essential as these elements are critical for various physiological processes, including vegetative growth (nitrogen), root development and flowering (phosphorus), overall plant vigor and disease resistance (potassium), and enzymatic activities and chlorophyll formation (zinc and copper) [24–26].

The leaf chlorophyll content was measured over two consecutive growing seasons (2023 and 2024) via a Minolta SPAD chlorophyll meter (Model No. 502) following Yadava [27] to assess treatment performance under various climatic conditions. At the end of the first season (2023), leaf nutrients analysis (N, P, K, Zn, and Cu) was conducted at the Alexandria University Facility, to establish a baseline for the nutritional response of the plants. Owing to the resource-intensive nature of these analyses, a single-season (2023) assessment was performed to provide a mechanistic understanding of treatment effects on nutrient status rather than seasonal dynamics.

Nitrogen was determined from dried leaf samples (70 °C, constant weight) digested with H₂SO₄ and H₂O₂, following Evenhuis and De-Waard [28, 29]. For the P and K contents, the dried leaves were placed in a muffle furnace at 550 °C. The ash was then dissolved in 2 N HNO_3_ [30]. The phosphorus content in the solution was determined spectrophotometrically at 470 nm via the vanadate molybdate method, whereas the potassium content was measured via a flame photometer [30]. Finally, the Zn and Cu contents were determined via a Perkin-Elmer Atomic Absorption Spectrophotometer [31].

### Statistical analysis

All data were analyzed using two-way analysis of variance (ANOVA) to determine the effects of light regime, foliar spray treatment, and their interaction as described by Gomez and Gomez [32].

We first employed ANOVA to ascertain whether significant differences existed among the group averages. If the overall ANOVA test indicated a statistically significant difference (*p* < 0.05), further pairwise comparisons were conducted. The ANOVA model included light regime, foliar spray treatment, and their interaction as fixed effects, while experimental error was considered random. Interaction effects were evaluated prior to interpretation of main effects, and when significant interactions were detected, treatment means were compared within each level of the interacting factor. When significant effects were detected, treatment means were separated using the least significant difference (LSD) test at *P* ≤ 0.05. The LSD₀.₀₅ value represented the minimum difference between two means required to conclude, with 95% confidence, that those two groups were truly distinct and not merely different due to random chance. Prior to analysis, data were checked for normality and homogeneity of variance using residual diagnostic plots. When necessary, data were transformed to meet ANOVA assumptions. All statistical analyses were performed using SAS software.

## Results

**Fig. 3:**
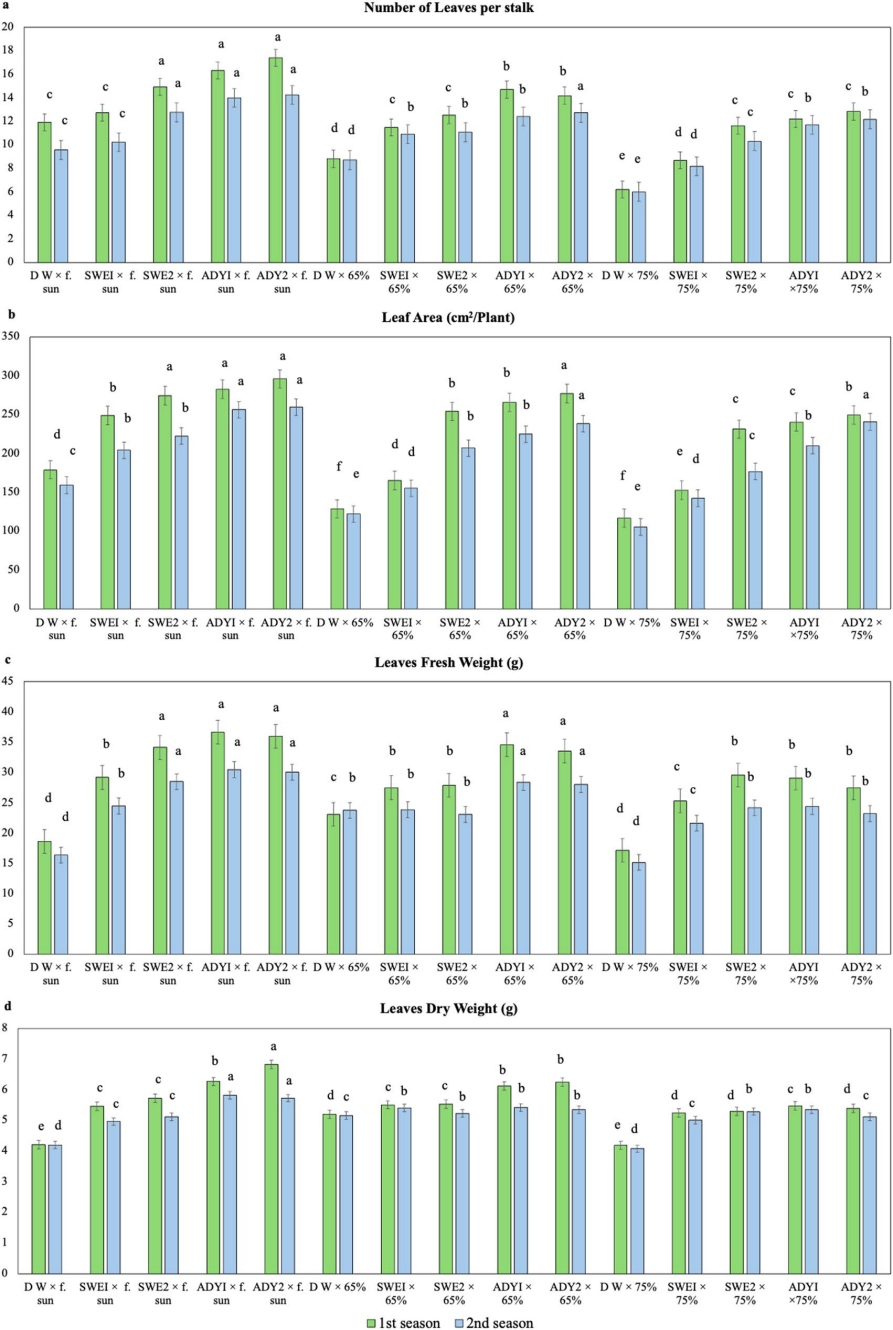
Interaction effects of light intensity and different concentrations of biostimulants on the vegetative growth and chlorophyll content of *R. hybrida.* LSD 0.05 = List significant differences at 0.05 probability. Means with the same letters are not significantly different at *p* ≤0.05 according to Fisher’s LSD test, where full sun, 65%, and 75% are the three different light intensities. DW, SWEI, SWE2, ADYI, and ADY2 are the five foliar spray treatments with distilled water, seaweed concentrations of 1000 and 2000, and active dried yeast concentrations of 3000 and 4000 mg. L^− 1,^ respective. The error bars represent the standard error (SE)

**Fig. 4:**
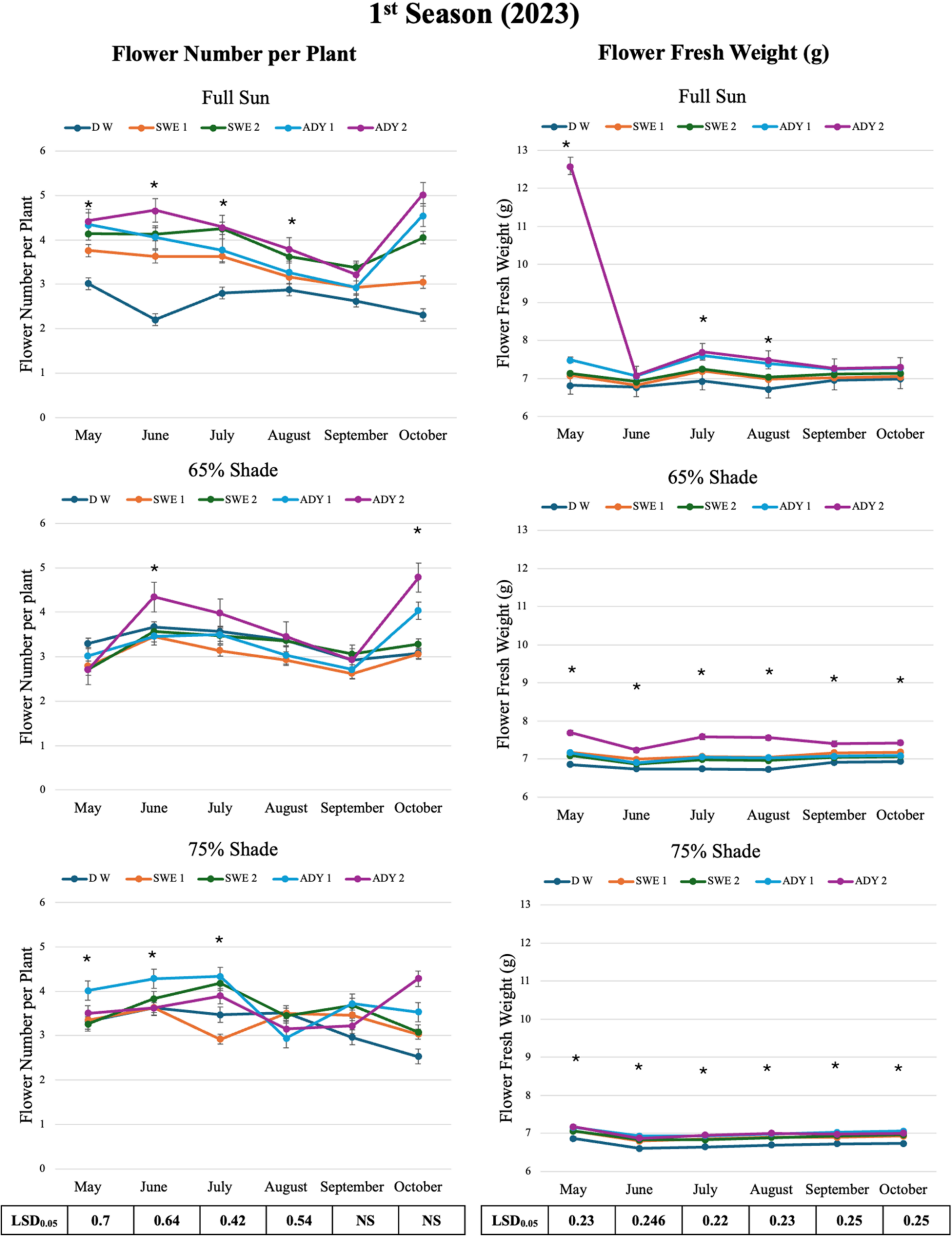
Interaction effects of light intensity and different concentrations of biostimulants on the flower productivity of *R. hybrida* during the 2023 season (May-October). LSD = List significant differences at 0.05 probability. Each value represents the mean of five biological replicates (*n* = 5), where one plant was considered as an experimental unit. Means with the asterisk (*) indicates significant differences at *p* ≤ 0.05 according to Fisher LSD test, within the same light regime, where full sun, 65%, and 75% are the three different light intensities. DW, SWEI, SWE2, ADYI, and ADY2 are the five foliar spray treatments with distilled water, seaweed concentrations of 1000 and 2000, and active dried yeast concentrations of 3000 and 4000 mg L^-1,^ respectively. The error bars represent the standard error (SE)

The results of the two-way analysis of variance (ANOVA) indicated significant main effects of light intensity and foliar spray treatments, as well as significant interaction effects for many measured parameters. In the following sections, Tables 2, 3, and 4 present the marginal means for the main effects of light intensity (averaged across all foliar spray treatments) and foliar spray (averaged across all light regimes). The detailed interaction effects, illustrating the performance of each specific treatment combination (Light × Foliar Spray), are graphically represented in Figs. 3, 4, 6, 7, and 8. This approach allows for a clear understanding of both individual factor influences and their synergistic or antagonistic interactions.

**Table 2:** Effects of light intensity, seaweed extract, and active dry yeast on the vegetative growth parameters and chemical composition of Rose plants during the 2023 and 2024 seasons. Two factor RCBD: factor A = light (full sun, 65%, 75% shade), factor B = foliar spray (DW, SWE1000, SWE2000, ADY3000, ADY4000). Main effect means are marginal means over the other factor

Treatments	Parameter	Number of leaves per stalk		Leaf area (cm^2^)		Leaves fresh weight (g)		Leaves Dry weight (g)		Chlorophyll content (SPAD values)	
	Season	1st season	2nd season	1st season	2nd season	1st season	2nd season	1st season	2nd season	1st season	2nd season
Light Intensity	Full sun	14.66 ± 1.04 a	12.16 ±0.96 a	256.11 ±20.8 a	220.36 ±18.5 a	30.96 ±3.3a	25.99±2.6a	5.7 ±0.4a	5.17 ±0.3b	56.52 ±1.9 a	45.22 ±1.5a
	65%	12.35 ±1.09 b	11.16 ±0.71 b	218.14±30.0 b	199.47 ±21.9 b	29.35 ±2.1a	25.431.1a	5.66 ±0.2a	5.31±0.1a	56.37 ±2.2a	45.1 ±1.6a
	75%	10.31 ±1.25 c	9.67 ±1.15 c	198.05 ±26.6 c	179.03 ±23.9 c	25.75 ±2.3b	21.731.7b	5.12 ±0.2b	4.97±0.3 c	54.25 ±0.6 b	43.4 ±0.5b
	LSD_0.05_	0.83	0.92	16.15	11.95	2.43	1.85	0.19	0.17	2	1.65
Foliar spray (mg. L^− 1^)	D W	8.98 ±1.65 d	8.09 ±1.07 d	141.43 ±19.0 d	128.95 ±15.9 e	21.29 ±1.8d	18.432.7d	4.53 ±0.3e	4.48 ±0.3d	44.46 ±2.0d	41.56 ±1.6d
	SWE 1000	10.96 ±1.2 c	9.77 ±0.82 c	188.84 ±30.2 c	167.23±18.8 d	27.36±1.1c	23.340.9c	5.4 ±0.1c	5.13 ±0.1c	47.41 ±1.2c	43.92 ±1.0c
	SWE 2000	13.04 ±0.98 b	11.38 ±0.72 b	253.26 ±12.5 b	202.05 ±13.4 c	30.57±1.9b	25.271.7b	5.52 ±0.1b	5.21 ±0.0b	49.05 ±1.7b	45.24 ±1.4b
	ADY 3000	14.41 ±1.2 a	12.7 ±0.68 a	262.9 ±12 ab	230.31±13.7 b	33.49±2.3a	27.771.8a	5.96 ±0.2a	5.52 ±0.1a	49.01 ±2.0b	45.21 ±1.5b
	ADY 4000	14.81 ±1.36 a	13.14 ±0.62 a	274.08 ±13.5 a	246.21 ±6.7 a	32.37±2.5a	27.132.0a	5.17 ±0.4d	5.43 ±0.2a	51.15 ±2.1a	46.92 ±1.8a
	LSD_0.05_	1.1	1.15	16.8	12.09	1.64	1.25	0.11	0.12	1.67	1.3

• Means (±SE) within a column followed by the same letter are not significantly different according to the LSD (*p* ≤ 0.05)

• Data for light Intensity are marginal means averaged across all foliar spraying treatments

• Data for foliar spraying are marginal means averaged across all light intensity conditions

• The values represent the means for the 2023 (1st season) and 2024 (2nd season) experiments

• The data presented in this table are marginal means, where values for Light Intensity are averaged across all foliar spray treatments, and values for Foliar Spray are averaged across all light intensity conditions. For the specific interaction effects (Light × Foliar Spray) and the performance of each treatment combination, please refer to Figs. 3 and 6

• *DW* distilled water, *SWE* seaweed extract, *ADY* active dry yeast

**Table 3:** Effects of light intensity and foliar sprays (seaweed extract, and active dry yeast) on the flower number of rose plants during the 2023 season. Two factor RCBD: factor A = light (full sun, 65%, 75% shade), factor B = foliar spray (DW, SWE1000, SWE2000, ADY3000, ADY4000). Main effect means are marginal means over the other factor

Treatments	Month	Flower number per plant					
		May	June	July	August	September	October
Light Intensity	Full sun	3.94 ±0.3 a	3.74±0.4 a	3.75±0.3 a	3.34±0.2 a	3.02±0.1 b	3.8±0.5 a
	65%	2.91±0.1 c	3.7±0.2 a	3.53±0.1 b	3.23±0.1 a	2.85±0.1 b	3.65±0.3 a
	75%	3.49±0.1b	3.8±0.1 a	3.76±0.3 a	3.31±0.1 a	3.41±0.1 a	3.29±0.3 b
	LSD_0.05_	0.12	NS	0.23	NS	0.24	0.23
Foliar spray (mg.L -1)	D W	3.21±0.1 b	3.17±0.5 c	3.28±0.2 b	3.25±0.2 a	2.84±0.1 b	2.64±0.2 c
	SWE 1000	3.3±0.3 b	3.57±0.1 b	3.23±0.2 b	3.2±0.2 a	3 ±0.2 b	3.05±0.0 bc
	SWE 2000	3.37±0.4 ab	3.84±0.2 ab	3.97±0.3 a	3.48±0.1 a	3.37±0.2 a	3.47±0.3 b
	ADY 3000	3.79±0.4 a	3.93±0.2 ab	3.86±0.2 a	3.08±0.1 a	3.12±0.3 ab	4.04±0.3 b
	ADY 4000	3.55±0.5 ab	4.21±0.3 a	4.05±0.1 a	3.46±0.2 a	3.12±0.1 ab	4.7±0.2 a
	LSD_0.05_	0.4	0.37	0.34	NS	0.26	0.62

• Means (±SE) within a column followed by the same letter within the same treatment group are not significantly different according to the LSD (*p* ≤ 0.05)

• The data presented in this table are marginal means, where values for Light Intensity are averaged across all foliar spray treatments, and values for Foliar Spray are averaged across all light intensity conditions. For the specific interaction effects (Light × Foliar Spray) and the performance of each treatment combination, please refer to Fig. 4

• *DW* distilled water, *SWE* seaweed extract, *ADY *active dry yeast

**Table 4:** Effects of light intensity and foliar sprays (seaweed extract, and active dry yeast) on the flower fresh weight (g) of rose plants during the 2023 season. Two factor RCBD: factor A = light (full sun, 65%, 75% shade), factor B = foliar spray (DW, SWE1000, SWE2000, ADY3000, ADY4000). Main effect means are marginal means over the other factor

Treatments	Flower fresh weight (g)					
	May	June	July	August	September	October
Light Intensity	8.22±1.1 a	6.93±0.1 a	7.33±0.1 a	7.12±0.1 a	7.12±0.2 a	7.15±0.2a
	7.19±0.1 b	6.95±0.1 a	7.08±0.1 b	7.06±0.1 a	7.12±0.1 a	7.14±0.1a
	7.06±0.1 c	6.8±0.1 b	6.84±0.1 c	6.89±0.1 b	6.91±1.0 b	6.93±0.1b
	0.12	0.08	0.12	0.1	0.08	0.08
Foliar spray (mg.L -1)	6.84±0.0 c	6.7±0.1 c	6.77±0.1 c	6.71±0.0 d	6.86±0.1 c	6.88±0.1 c
	7.1±0.0 b	6.87±0.1 bc	7.03±0.1 b	6.97±0.0 c	7.02±0.1 b	7.05±0.1 b
	7.09±0.0 b	6.87±0.0 bc	7.02±0.1 b	6.96±0.0 c	7.03±0.1 b	7.05±0.1 b
	7.27±0.1 b	6.96±0.1 ab	7.19±0.2 b	7.13±0.1 b	7.11±0.2 ab	7.14 ±0.2ab
	9.15±1.7 a	7.06±0.1 a	7.41±0.2 a	7.35±0.1 a	7.21±1.4 a	7.24±0.3 a
	0.27	0.15	0.14	0.12	0.14	0.14

• Means (±SE) within a column followed by the same letter within the same treatment group are not significantly different according to the LSD (*p* ≤ 0.05)

• The data presented in this table are marginal means, where values for Light Intensity are averaged across all foliar spray treatments, and values for Foliar Spray are averaged across all light intensity conditions. For the specific interaction effects (Light × Foliar Spray) and the performance of each treatment combination, please refer to Fig. 4

• *DW* distilled water, *SWE* seaweed extract, *ADY *active dry yeast

### Morphological trait under light intensity

Light intensity was the main factor governing vegetative performance in *R. hybrida.* Plants under full sun exhibited significantly higher total fresh and dry weights, leaf number, and leaf area compared to shaded plants (Table 2). As shade increased to 75%, these parameters declined markedly, confirming that reduced irradiance limited photosynthetic activity and biomass accumulation. The number of leaves per flowering stalk and leaf area likewise declined with increasing shade intensity. Mean values under full sun were 14.66–12.16 leaves per stalk and 256.11–220.36 cm²/plant across the two seasons, compared with only 10.31–9.67 leaves and 198.05–179.03 cm² under 75% shade. These data confirm that higher irradiance supports biomass accumulation and leaf expansion.

Having established that light intensity was the primary determinant of vegetative growth, the next step was to examine whether biostimulant applications could enhance or compensate for the effects observed under different light levels.

### Effects of biostimulants applications on vegetative growth

After establishing the baseline effect of light intensity, the influence of biostimulant applications was evaluated to determine how these treatments modified plant responses under different irradiance levels. Foliar sprays of SWE and ADY markedly enhanced vegetative traits compared with the distilled water control, particularly under full sun conditions. The results showed clear positive effects of the biostimulants (Table 2). Foliar applications of SWE (1000 and 2000 mg L^− 1^) and ADY (3000 and 4000 mg L^− 1^) produced marked improvements in vegetative traits (Table 2). Both products significantly increased the number of leaves per flowering stalk and leaf area per plant compared to the distilled water (DW) control. The effect was concentration dependent, with the highest increase recorded at 4000 mg L^− 1^ ADY 4000 treatments. The values for ADY4000 reached 14.81–12.97 leaves per flowering stalk and 274.08–246.21 cm^2^ per plant (first and second seasons, respectively), which represents a substantial improvement compared with those of the untreated control plants, which recorded only 8.98–8.09 leaves per flowering stalk and 141.43–128.95 cm^2^ per plant in the respective seasons (Table 2).

A strong interaction between light intensity and biostimulants concentration was noticeable. The combined application of ADY3000 or ADY4000 under full sun yielded the most pronounced morphological enhancement, indicating a synergistic effect between optimal irradiance and growth-promoting compounds. (Fig. 3). The improvements in vegetative growth prompted by light and biostimulant treatments were reflected in the flowering phase. Therefore, the following section evaluates how variations in light and foliar applications influenced flower production and quality.

### Flower productivity under varying light and foliar treatments

Flowering performance was primarily driven by light intensity, with the greatest flower number and fresh weight consistently recorded under full sun. Building upon this pattern, the application of SWE and ADY further improved flower production across light levels, with the strongest enhancement observed under full sun conditions (Tables 3 and 4, Fig. 4). A gradual increase in flower number per plant was observed with increasing concentrations of SWE and ADY in both growing seasons (Table 3). Under full sun, the flower number was significantly high in all months; for instance, the mean flower number in May of the first season reached 3.94 flowers per plant under full sun, whereas it was 2.91 under 65% shade (Fig. 4). This finding is supported by the observation of poor flowering in *R. hybrida* plants grown under shaded conditions, while planting under full sun increased flower quality. The ADY4000 × full sun combination produced the maximum flower number (4.67 in June, first season) (Fig. 4).

Flower fresh weight followed a similar trend (Table 4). The greatest mean fresh weight occurred under ADY 4000 x full sun (12.59 g) in May of the first season, while the DW control consistently exhibited the lowest value (6.84 g) (Fig. 4). Full sun and 65% shading performed similarly in the first season, but full sun was significantly superior in the second (Fig S1). Both SWE concentrations (1000 and 2000 mg L^− 1^) were similar in effect and significantly greater than those of the control, with ADY 4000 mg L^− 1^ outperformed ADY3000 in most months (Fig. 4 and S1).

The most pronounced effect on both flower number and fresh weight was the synergistic combination of ADY4000 x full sun, which resulted in high flower fresh weight of 12.59 g in May of the first season (Fig. 4). This overall increase in flower fresh weight with increasing SWE and ADY levels likely reflect the efficiency of available nutrients and photosynthetic products induced by biostimulants.

Because flower productivity is closely linked to stem development, the subsequent section focuses on how light intensity and biostimulant applications affected stalk length and thickness across seasons.

### Stalk length and thickness responses

**Fig. 5:**
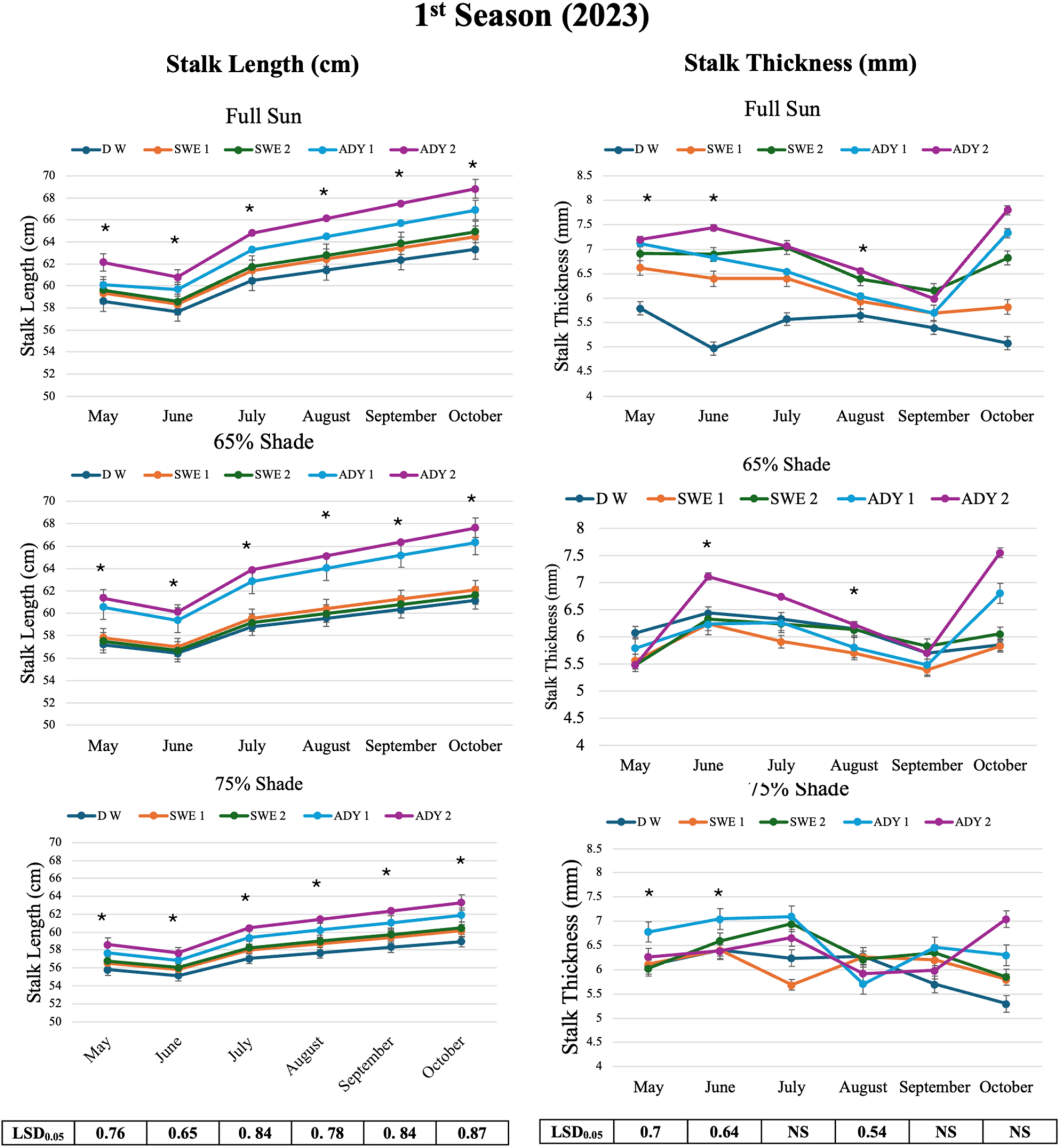
Interaction effects of light intensity and different concentrations of biostimulants on the stalk quality of *R. hybrida* during the 2023 season (May-October). LSD = List significant differences at 0.05 probability. Each value represents the mean of five biological replicates (*n* = 5), where one plant was considered as an experimental unit. Means with the asterisk (*) indicates significant differences at *p* ≤ 0.05 according to Fisher LSD test, within the same light regime, where full sun, 65%, and 75% are the three different light intensities. DW, SWEI, SWE2, ADYI, and ADY2 are the five foliar spray treatments with distilled water, seaweed concentrations of 1000 and 2000, and active dried yeast concentrations of 3000 and 4000 mg L^-1,^ respectively. The error bars represent the standard error (SE)

Variations in light intensity established clear differences in stalk elongation and stem thickening, with full sun promoting superior structural growth across seasons. The addition of foliar biostimulants, particularly ADY4000, amplified these effects and produced the tallest and thickest stalks when applied under optimal light conditions (Tables 5 and 6, Fig. 5). The effects of varying light intensities and foliar spray treatments on stalk traits were evaluated to assess flower quality over two growing seasons (Table 5). The combined application of ADY4000 x full sun consistently resulted in the greatest stalk length and thickness, reaching the highest values of 68.8 cm and 7.8 mm, respectively, in October of the second season (Fig. 5). Conversely, the DW x 75% shading treatment presented the poorest growth, with the stalk length and stem thickness reduced to 55.1 cm and 4.3 mm, respectively (Fig. 5). Across treatments, full sun was consistently associated with enhanced growth compared with 65% and 75% shading. Moderate shade (65%) maintained acceptable growth only when high ADY concentrations (3000–4000 mg L⁻¹) were applied (Fig. 5 and S2).

**Table 5:** Effects of light intensity and foliar sprays (seaweed extract, and active dry yeast) on the stalk length (cm) of rose plants during the 2023 season. Two factor RCBD: factor A = light (full sun, 65%, 75% shade), factor B = foliar spray (DW, SWE1000, SWE2000, ADY3000, ADY4000). Main effect means are marginal means over the other factor

Treatments	Month	Stalk length (cm)					
		May	June	July	August	September	October
Light Intensity	Full sun	59.96±0.6 a	59.01±0.6 a	62.35±0.8 a	63.46±0.8 a	64.58±0.9 a	65.69±1.0 a
	65%	58.89±0.9 b	57.91±0.8 b	60.84±1.0 b	61.81±1.1 b	62.79±1.2 b	63.77±1.3 b
	75%	57.08±0.5 c	56.3±0.4 c	58.63±0.6 c	59.4±0.6 c	60.18±0.7 c	60.96±0.8 c
	LSD_0.05_	0.63	0.62	0.8	0.77	0.82	0. 89
Foliar spray (mg.L^− 1^)	D W	57.2±0.8 d	56.41±0.7d	58.77±1.0 d	59.56±1.1 d	60.35±1.2 d	61.14±1.3 d
	SWE 1000	57.91±0.8 c	57.04±0.7 c	59.65±1.0 c	60.52±1.1 c	61.38±1.2 c	62.25±1.3 c
	SWE 2000	57.96±0.9 c	57.09±0.8 c	59.71±1.0 c	60.59±1.1 c	61.46±1.2 c	62.34±1.3 c
	ADY 3000	59.44±0.9 b	58.64±0.9 b	61.84±1.2 b	62.91±1.4 b	63.981.5 b	65.05±1.6 b
	ADY 4000	60.7 ±1.1a	59.52±1.0 a	63.05±1.3 a	64.23±1.4 a	65.4±1.6 a	66.59±1.7 a
	LSD_0.05_	0.43	0.44	0.52	0.62	0.65	0.61

• Means (±SE) within a column followed by the same letter within the same treatment group are not significantly different according to the LSD (*p* ≤ 0.05)

• The data presented in this table are marginal means, where values for Light Intensity are averaged across all foliar spray treatments, and values for Foliar Spray are averaged across all light intensity conditions. For the specific interaction effects (Light × Foliar Spray) and the performance of each treatment combination, please refer to Fig. 5

• *DW* distilled water, *SWE* seaweed extract, *ADY *active dry yeast

**Table 6:** Effects of light intensity and foliar sprays (seaweed extract, and active dry yeast) on the stalk thickness (mm) of rose plants during the 2023 season. Two factor RCBD: factor A = light (full sun, 65%, 75% shade), factor B = foliar spray (DW, SWE1000, SWE2000, ADY3000, ADY4000). Main effect means are marginal means over the other factor

Treatments	Stalk Thickness (mm)					
	May	June	July	August	September	October
Light Intensity	6.73±0.3 a	6.51±0.4 a	6.52±0.3 a	6.11±0.2 a	5.79±0.1 ab	6.57±0.5 a
	5.68±0.1 b	6.47±0.2 a	6.3±0.1 a	6:00±0.1 a	5.62±0.1 b	6.42±0.3 a
	6.26±0.1 c	6.57±0.1 a	6.53±0.3 a	6.08±0.1 a	6.14±0.1 a	6.06±0.3 b
	0.34	NS	NS	NS	0.41	0.37
Foliar spray (mg.L^− 1^)	5.98±0.1 b	5.94±0.5 d	6.05±0.2 c	6.02±0.2 bc	5.6±0.1 c	5.41±0.2 e
	6.1±0.3 b	6.34±0.1 c	6±0.2 c	5.97± 0.2bc	5.76±0.2 bc	5.82±0.0 d
	6.14±0.4 b	6.61±0.2b	6.74±0.3 ab	6.25 ±0.1a	6.11±0.2a	6.24±0.3 c
	6.56±0.4 a	6.7±0.2 b	6.63±0.2 b	5.85±0.1 c	5.88±0.3 ab	6.81±0.3 b
	6.32±0.5 b	6.98±0.3 a	6.82±0.1 a	6.23±0.2 ab	5.89±0.1 ab	7.47±0.2 a
	0.22	0.21	0.17	0.27	0.24	0.21

• Means (±SE) within a column followed by the same letter within the same treatment group are not significantly different according to the LSD (*p* ≤ 0.05)

• The data presented in this table are marginal means, where values for Light Intensity are averaged across all foliar spray treatments, and values for Foliar Spray are averaged across all light intensity conditions. For the specific interaction effects (Light × Foliar Spray) and the performance of each treatment combination, please refer to Fig. 5

• *DW* distilled water, *SWE* seaweed extract, *ADY *active dry yeast

These findings emphasize the synergistic effects of optimal light conditions and nutrient-enriched foliar sprays in enhancing structural development. Overall, the results demonstrate that the application of high-concentration ADY under full sun provides the most favorable conditions for maximizing stalk and flower stem growth across both growing seasons.

To better understand the physiological basis for the observed morphological and flowering responses, chlorophyll levels and nutrient concentrations were assessed under the same treatment conditions.

### Chlorophyll and nutrients content responses

**Fig. 6:**
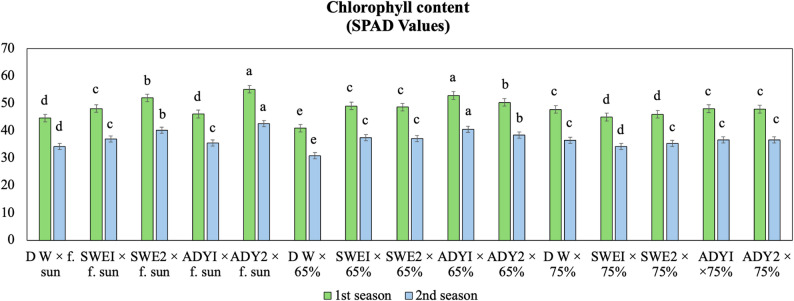
Interaction effects of light intensity and different concentrations of biostimulants on the chlorophyll content of *R. hybrida.* LSD 0.05 = List significant differences at 0.05 probability. Means with the same letters are not significantly different at *p* ≤0.05 according to Fisher’s LSD test, where full sun, 65%, and 75% are the three different light intensities. DW, SWEI, SWE2, ADYI, and ADY2 are the five foliar spray treatments with distilled water, seaweed concentrations of 1000 and 2000, and active dried yeast concentrations of 3000 and 4000 mg. L^− 1,^ respective. The error bars represent the standard error (SE)

**Fig. 7:**
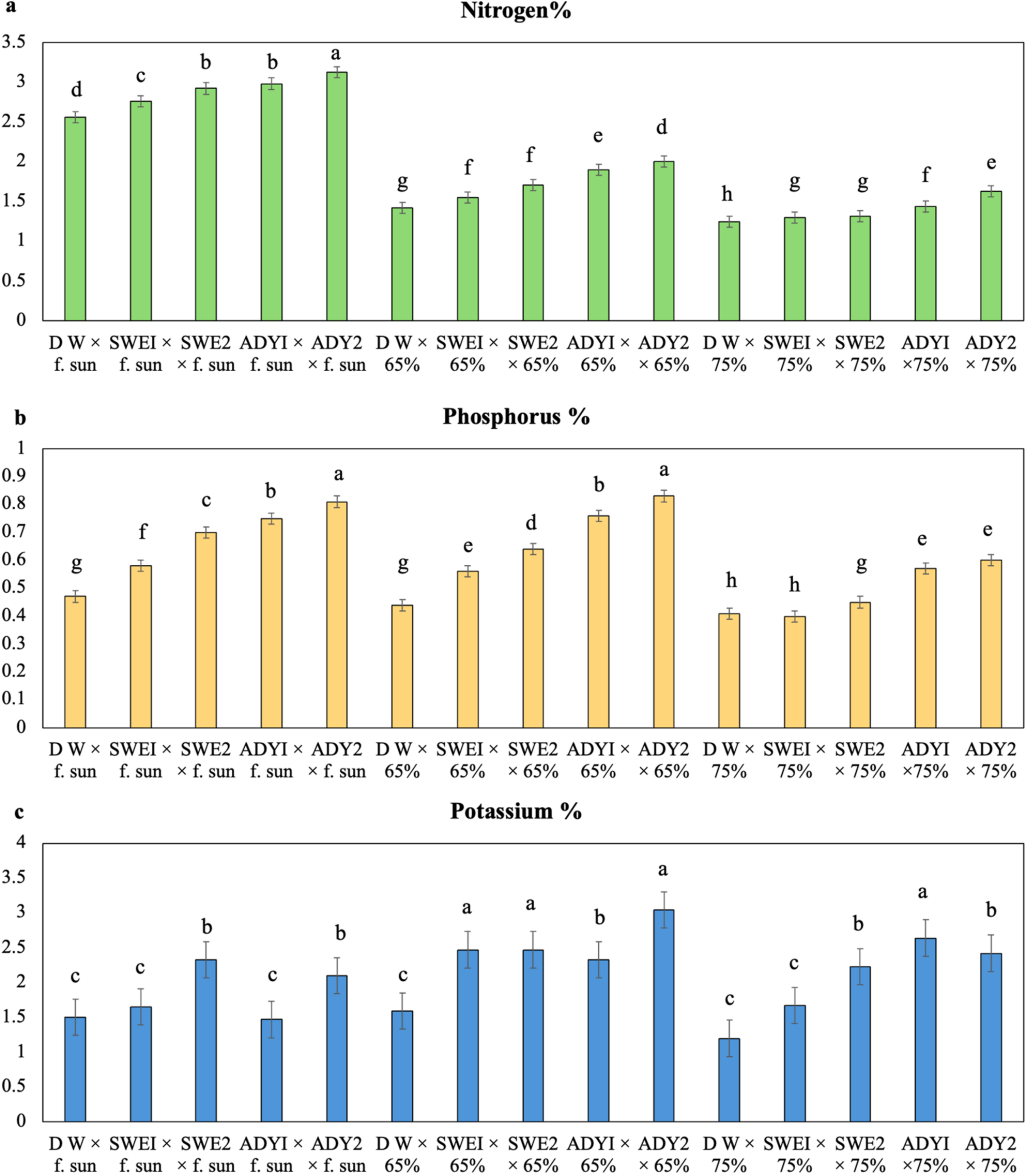
Interaction effects of light intensity and different concentrations of biostimulants on the macronutrient composition (%) of the leaves of *R. hybrida* during the 2023 season. LSD 0.05 = List significant differences at 0.05 probability. Means with the same letters are not significantly different at *p* ≤ 0.05 according to Fisher’s LSD test, where full sun, 65%, and 75% are the three different light intensities. DW, SWEI, SWE2, ADYI, and ADY2 are the five foliar spray treatments with distilled water, seaweed concentrations of 1000 and 2000, and active dried yeast concentrations of 3000 and 4000 mg. L^− 1,^ respectively. The error bars represent the standard error (SE)

**Fig. 8:**
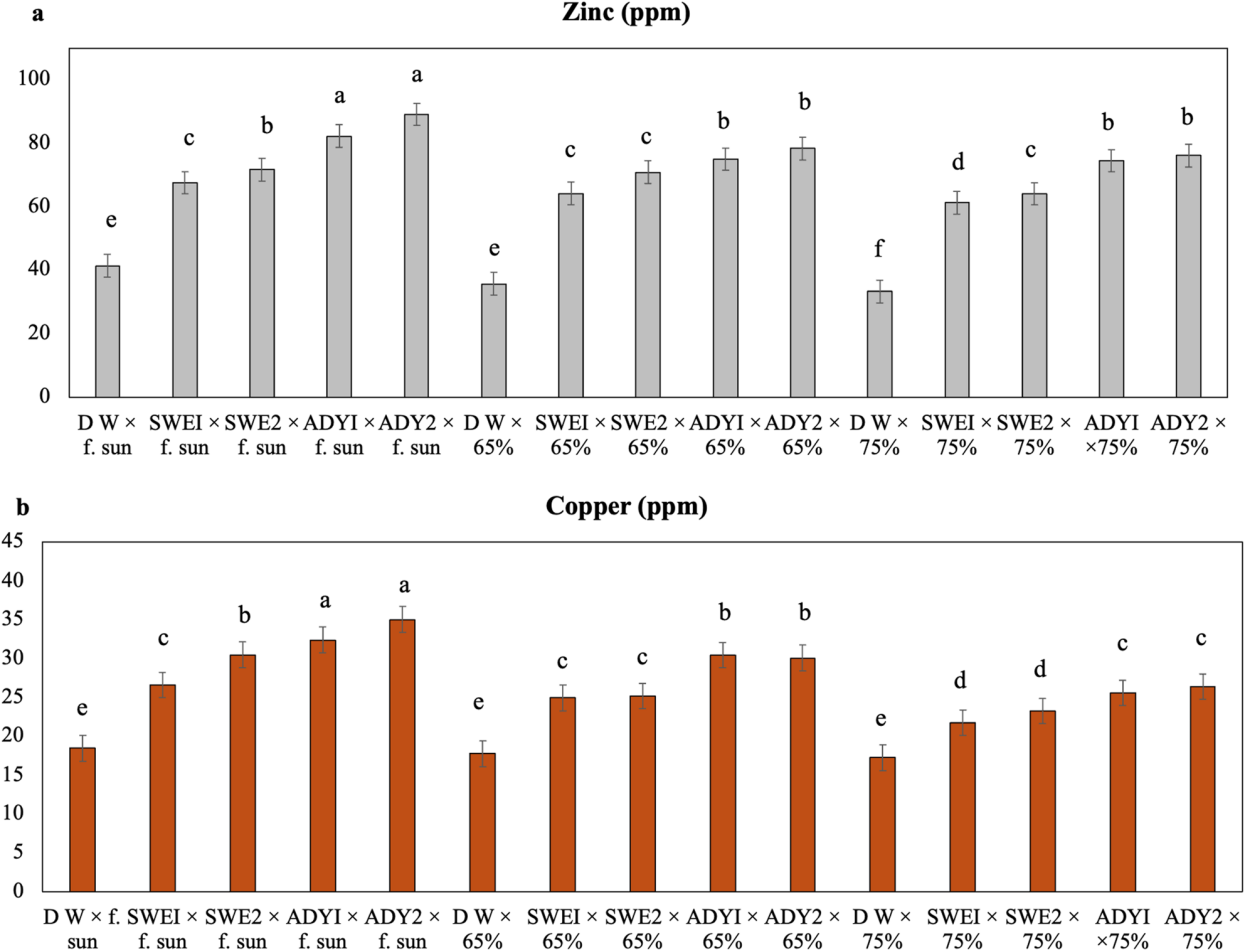
Interaction effects of light intensity and different concentrations of biostimulants on the zinc and copper composition (ppm) of the leaves of *R. hybrida* during the 2023 season. LSD 0.05 = List significant differences at 0.05 probability. Means with the same letters are not significantly different at *p* ≤ 0.05 according to Fisher’s LSD test, where full sun, 65%, and 75% are the three different light intensities. DW, SWEI, SWE2, ADYI, and ADY2 are the five foliar spray treatments with distilled water, seaweed concentrations of 1000 and 2000, and active dried yeast concentrations of 3000 and 4000 mg. L^− 1,^ respectively. The error bars represent the standard error (SE)

Light intensity significantly influenced chlorophyll content and nutrient accumulation, confirming its primary role in determining photosynthetic capacity and leaf nutritional status. Under these established light conditions, foliar applications of ADY and SWE further enhanced chlorophyll values and nutrient uptake, particularly in plants grown under full sun (Tables 2 and 7, Figs. 6, 7, and 8). In the first season, light intensity was a critical factor, with full sun and 65% light conditions yielding the highest chlorophyll content, with statistically similar mean SPAD values of 56.52 and 56.37, respectively, which were significantly greater than the 54.25 SPAD value observed under 75% shade (Table 2, Fig. 6).

**Table 7:** Effects of light intensity, seaweed extract, and active dry yeast on the chemical composition of the leaves of rose plants during the 2023 season. Two factor RCBD: factor A = light (full sun, 65%, 75% shade), factor B = foliar spray (DW, SWE1000, SWE2000, ADY3000, ADY4000). Main effect means are marginal means over the other factor

Treatments	Parameter	Nitrogen%	Phosphorus %	Potassium %	Zn (ppm)	Cu (ppm)
Light Intensity	Full sun	2.87 ±0.1 a	0.66±0.06 a	1.81±0.17 b	70.43±8.19 a	28.63±2.88 a
	65%	1.32±0.09 b	0.65±0.06 a	2.38±0.19 a	64.867.93 b	25.71±2.33 b
	75%	1.39±0.07 b	0.49±0.04 b	2.03±0.26 b	61.927.69 b	22.87±1.63 c
	LSD_0.05_	0.28	0.02	0.29	4.18	2.36
Foliar spray (mg. L^− 1^)	D W	1.74±0.41 d	0.44±0.02 e	1.43±0.12 c	36.85±2.37 d	17.86±0.36 c
	SWE 1000	1.87±0.45 c	0.51±0.06 d	1.93±0.27 b	64.37±1.81c	24.45±1.43 b
	SWE 2000	1.98±0.48 c	0.59±0.08 c	2.34±0.07 ad	68.93±2.41 b	26.34±2.16 b
	ADY 3000	2.11±0.46 b	0.69±0.06 b	2.15±0.35 b	71.29±2.51 b	29.50±2.02 a
	ADY 4000	2.29±0.45 a	0.75±0.07 a	2.52±0.28 a	81.25±3.98 a	30.52±2.5 a
	LSD_0.05_	0.08	0.03	0.34	3.08	2.81

• Means (±SE) within a column followed by the same letter within the same treatment group are not significantly different according to the LSD test (*p* ≤ 0.05)

• Data for Light Intensity are marginal means averaged across all foliar spraying treatments

• Data for Foliar Spraying are marginal means averaged across all light intensity conditions

• The data presented in this table are marginal means, where values for Light Intensity are averaged across all foliar spray treatments, and values for Foliar Spray are averaged across all light intensity conditions. For the specific interaction effects (Light × Foliar Spray) and the performance of each treatment combination, please refer to Figs. 7 and 8

• *DW* distilled water, *SWE* seaweed extract, *ADY *active dry yeast

To understand the nutritional basis for the observed differences in rose growth and chlorophyll content, the concentrations of key macro- and micronutrients in the leaf tissue were determined for the 2023 season. (Table 7, Figs. 7 and 8). The results indicate that both the light intensity and foliar spray treatments significantly influenced nutrient accumulation. Under full sun, the plants presented the highest concentrations of N (2.87%), P (0.66%), Zn (70.43 ppm), and Cu (28.63 ppm), significantly exceeding those under 65% and 75% shade. In contrast, the K concentration was highest under the 75% shade treatment (2.38%) (Table 7). Among foliar sprays, ADY 4000 resulted in the highest N (2.29%), P (0.75%), Zn (81.25 ppm), and Cu (30.52 ppm) concentrations and ranked among the highest for K (2.52%). The distilled water (DW) control resulted in the lowest values across all nutrients.

A significant interaction effect between foliar spray and light intensity was detected (Figs. 7 and 8). Nutrient concentrations generally decreased with increasing shade. The ADY4000 treatment produced the highest N (3.12%), Zn (89.1 ppm), and Cu (35.05 ppm) levels, whereas the 65% shade treatment resulted in the highest P (0.83%) and K (3.04%) contents. Across all the light conditions, the nutrient accumulation in the ADY and SWE treatments was greater than that in the DW control, which, under 75% shade, presented the lowest nutrient concentrations. These findings highlight that the combined effects of adequate light and bio-stimulant application substantially improve chlorophyll content and nutrient accumulation, thereby supporting enhanced vegetative and reproductive growth.

## Discussion

The present study demonstrated that both light intensity and foliar application of biostimulants are key determinants of growth, productivity, and physiological performance in *R. hybrida*, as reflected in the pronounced effects on vegetative traits, flower yield, stalk quality, and nutrient status described in the Results. The consistent superiority of full sun plants, together with the strong enhancement by high-concentration ADY 4000, indicates that environmental and nutritional factors act in concert rather than independently. These integrated responses emphasize that optimizing light conditions is a prerequisite for fully realizing the growth-promoting potential of biostimulants in commercial rose production.

The cultivation of high-quality ornamental crops such as *R. hybrida* requires a clear understanding of the interplay between environmental factors and horticultural inputs. The findings of this study demonstrated that light intensity is a primary determinant of rose growth, productivity, and physiological health. Our observation that full sun exposure maximized morphological traits, including leaf biomass, leaf area, and flower number, is consistent with a large group of studies demonstrating that shading limits photosynthetic capacity, thereby reducing the energy available for growth and development. The significant inhibition of these parameters under 75% shading aligns with foundational work showing that insufficient light reduces photosynthetic activity, thereby limited carbohydrate production and mineral uptake, consequently lowering leaf fresh and dry biomass [33–37].

The central novelty of this research lies in elucidating the powerful, synergistic relationship between light availability and the application of biostimulants. While the growth-promoting properties of ADY and SWE are well documented [36, 38, 39], our study indicated that their efficacy is highly dependent on the ambient light environment. The most improvements in vegetative growth, flower number, and overall quality were consistently obtained when combined application of high-concentration ADY 4000 mg L⁻¹ was applied under full sun. These findings suggest that the rich combination of nutrients, amino acids, and phytohormones supplied by ADY is utilized most effectively when the plant’s photosynthetic machinery is operating at maximum capacity. Under full sun conditions, and SWE or control (DW), ADY-treated plants exhibited superior morphological performance, strongly suggesting the stimulatory role of yeast-derived nutrients and bioactive compounds in promoting cell division, elongation, and differentiation [40–42]. This response is likely related, at least in part, to the role of SWE and/or ADY under full sun in the synthesis of protein and cytokinin, consequently affecting cell division for flower initiation and development [36, 38]. This synergy refines our understanding that biostimulants function primarily as enhancers of plants already primed for growth rather than as a rescue treatment for poor conditions.

In this study, chlorophyll and key nutrients content analysis provided a strong mechanistic basis for these observations. The increased chlorophyll content under optimal light and biostimulants application is a key indicator of improved plant health. Light is a primary driver of chlorophyll synthesis and the observed increase is expected [43]. More importantly, the application of ADY and SWE further increased chlorophyll levels, likely through distinct but complementary mechanisms. Cytokinins, including those produced by yeast and applied exogenously to plants, can delay chlorophyll degradation (senescence) and promote RNA and protein synthesis [44, 45]. This effect was also noted by Hellal et al. (2011) in dill plants [46]. Similarly, betaines in SWE can reduce chlorophyll breakdown [47], supporting our findings and those on other ornamentals [48–50].

Furthermore, the enhanced nutritional status of plants treated with ADY under full sun directly explains the observed improvements in growth. These plants presented the highest concentrations of nitrogen, phosphorus, zinc, and copper, nutrients fundamental to plant function [51, 52]. Interestingly, the highest potassium concentration was recorded under 75% shade, which aligns with previous findings suggesting that shading may induce a regulatory shift in potassium transporter gene expression, promoting greater potassium retention in leaves [53]. This aligns with previous work showing that shade can limit nutrient absorption [54], whereas biostimulants can improve nutrient mobilization and uptake [55, 56]. The ability of ADY to increase the uptake of elements such as zinc explains its profound effect on plant vitality, as observed in previous studies [57]. This finding is consistent with a broad range of studies on ornamental species that have documented similar positive effects of biostimulants on nutrient content [50, 58, 59]. However, the specific role of ADY in increasing copper uptake has been less well documented in plant studies, and some studies have even suggested that certain yeasts might inhibit metal uptake to protect plants from toxicity. There is limited documentation to support a general role for ADY in increasing plant copper uptake in natural settings [60].

The practical implications of these findings are significant for commercial horticulture. Our results suggest that applying biostimulants to heavily shaded plants, while offering some marginal benefits, yields a diminished return on investment. The most effective and efficient strategy is to use these products to augment the performance of plants grown under optimal light. This integrated approach, which ensures optimal light conditions before the application of biostimulants, provides a precise and sustainable cultivation strategy, aligning with evidence from other species that highlights the importance of such interactions [16–19].

This study opens several important avenues for future research. While we have established a strong physiological link between light, biostimulants, and plant performance, a deeper molecular investigation is needed to identify the specific genes and signalling pathways coregulated by these factors. Furthermore, given the seasonal variations observed in our data, long-term trials across diverse climatic zones are necessary to validate the robustness of these findings and develop climate-resilient cultivation protocols. Finally, future work should incorporate a comprehensive economic analysis and explicit dose-response assessment of biostimulants application to evaluate the cost‒benefit ratio of high-concentration treatments and to define application rates that are both biologically and commercially viable for growers worldwide.

## Conclusion

This study investigated the synergistic effects of light intensity and biostimulants application on the growth, flowering, chlorophyll, and nutritional content of *Rosa hybrida* L. (Roses), providing a preliminary guidance for optimizing their cultivation under tested conditions. Within the limits of this experiment, the results clearly demonstrate that both factors are critical determinants of plant performance, with their combined application yielding the most significant improvements. The findings strongly support the hypothesis that optimizing roses cultivation requires a dual approach, with the combination of full sun and foliar application of ADY 4000 mg L^− 1^ represents the most effective management strategy for maximizing both yield and quality under tested conditions. Specifically, full sun conditions were consistently superior for maximizing vegetative growth, flower productivity, and the accumulation of key macronutrients (N and P). Thus, the application of ADY at 4000 mg L^− 1^ emerged as the most effective biostimulants significantly increasing all measured parameters, including the number of leaves per stalk, leaf area, flower number, flower fresh weight, chlorophyll content, and leaf N, P, and K percentages. The synergistic interaction between optimal light and the highest concentration of ADY resulted in the superior performance across all categories, such as the highest flower fresh weight and greater stalk quality. Conversely, 75% shading significantly inhibited growth and nutrient accumulation, even when combined with biostimulants.

**Fig. 9:**
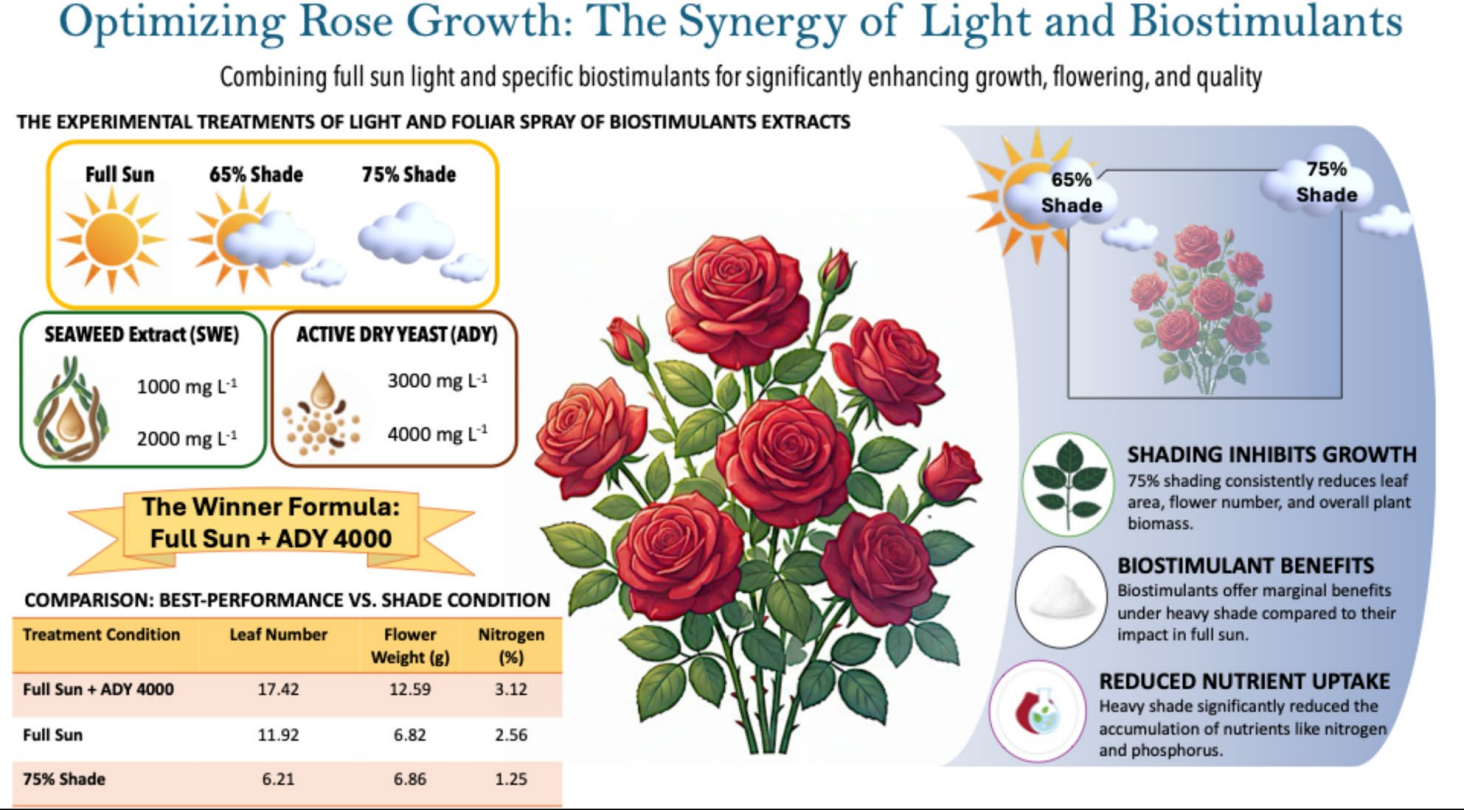
Schematic overview of the experimental design and key outcomes showing the combined effects of light intensity (full sun, 65% shade, and 75% shade) and foliar-applied biostimulants—seaweed extract (SWE; 1000 and 2000 mg L⁻¹) and active dry yeast (ADY; 3000 and 4000 mg L⁻¹)—on growth and nutrient status of Rosa hybrida L. The combination of full sun and ADY at 4000 mg L⁻¹ produced the highest leaf number, flower weight, and nitrogen content, whereas heavy shading (75%) reduced growth and nutrient accumulation

On this basis, the present results indicate that, for the conditions tested here, combined management with full sun exposure and foliar application of ADY 4000 mg L⁻¹ may be a promising strategy to improve growth and flowering of *R. hybrida* (Fig. 9). However, these conclusions are specific to the environmental conditions, cultivar, and management regime used in this study. Additional trials under diverse commercial production systems, cultivars, climates, and economic scenarios will be necessary to confirm these responses and to determine whether this combination represents a broadly applicable recommendation for rose growers.

## Supplementary Information

Supplementary Material 1.

## Data Availability

All the data generated or analysed during this study are included in this published article.
