# Supplementary material for: Optimizing the growth and flowering of Rosa hybrida L. (Roses) through synergistic light and biostimulant management
Source: BMC Plant Biol. 2026 May 26;26:917. doi: 10.1186/s12870-026-09034-3 (PMC13202870; doi:10.1186/s12870-026-09034-3)
Supplement: Supplementary file 1 — Supplementary Material 1. [file 12870_2026_9034_MOESM1_ESM.docx]

**Table S1** Effects of light intensity and foliar sprays (seaweed extract, and active dry yeast) on the flower number of rose plants during the 2024 season. Two factor RCBD: factor A = light (full sun, 65%, 75% shade), factor B = foliar spray (DW, SWE1000, SWE2000, ADY3000, ADY4000). Main effect means are marginal means over the other factor.

|  |  | **Flower number per plant** | | | | | |
| --- | --- | --- | --- | --- | --- | --- | --- |
| Treatments | **Month** | **May** | **June** | **July** | **August** | **September** | **October** |
| Light Intensity | **Full sun** | 3.3 ±0.4 a | 3.45±0.2 a | 3.62±0.3 a | 2.88±0.1 a | 2.84±0.1 b | 2.91±0.3 b |
|  | **65%** | 2.74±0.2 c | 3.38±0.1 a | 3.05±0.1 b | 2.85±0.1 a | 2.62±0.1 c | 3.13±0.2 a |
|  | **75%** | 3.08±0.1 b | 3.44±0.1 a | 3.61±0.1 a | 3.24±0.1 a | 3.17±0.1 a | 2.88±0.1 b |
|  | **LSD_0.05_** | 0.13 | NS | 0.2 | NS | 0.21 | 0.15 |
| Foliar spray (mg.L -1) | **D W** | 2.51±0.5 c | 3.18±0.2 a | 2.96±0.3 b | 3.02±0.1 a | 2.66±0.1 c | 2.4±0.3 d |
|  | **SWE 1000** | 2.94±0.3 b | 3.25±0.0 a | 3.43±0.5 ab | 2.86±0.2 a | 2.76±0.3 bc | 2.86±0.1 c |
|  | **SWE 2000** | 2.96±0.4 b | 3.47±0.2 a | 3.59±0.3 a | 3.08±0.1 a | 3.13±0.1 a | 3.1±0.2 b |
|  | **ADY 3000** | 3.3±0.4 ab | 3.49±0.2 a | 3.46±0.2 ab | 2.96±0.4 a | 2.91±0.2 ab | 3.38±0.1 a |
|  | **ADY 4000** | 3.47±0.3 a | 3.72±0.3 a | 3.7±0.1 a | 3.03±0.1 a | 2.92±0.1 ab | 3.13±0.3 b |
|  | **LSD_0.05_** | 0.4 | NS | 0.35 | NS | 0.24 | 0.2 |

- Means (±SE) within a column followed by the same letter within the same treatment group are **not significantly different** according to the LSD (p ≤ 0.05).
- The data presented in this table are **marginal means**, where values for Light Intensity are averaged across all foliar spray treatments, and values for Foliar Spray are averaged across all light intensity conditions. **For the specific interaction effects (Light × Foliar Spray) and the performance of each treatment combination, please refer to** Figure S1
- DW = distilled water; SWE = seaweed extract; ADY=active dry yeast.

**Table S2** Effects of light intensity and foliar sprays (seaweed extract, and active dry yeast) on the flower fresh weight (g) of rose plants during the 2024 season. Two factor RCBD: factor A = light (full sun, 65%, 75% shade), factor B = foliar spray (DW, SWE1000, SWE2000, ADY3000, ADY4000). Main effect means are marginal means over the other factor.

|  |  | **Flower fresh weight (g)** | | | | | |
| --- | --- | --- | --- | --- | --- | --- | --- |
| Treatments | **Month** | **May** | **June** | **July** | **August** | **September** | **October** |
| Light Intensity | **Full sun** | 7.03±0.1 a | 6.77±0.1 a | 6.63±0.1 a | 6.43±0.1 a | 7.41±0.2 b | 7.55±0.2 a |
|  | **65%** | 7:00±0.1 a | 6.66±0.1 b | 6.49±0.1 b | 6.32±0.1 b | 7.17±0.1 c | 7.35±0.1b |
|  | **75%** | 6.88±0.0 b | 6.49±0.0 c | 6.33±0.0 c | 6.25±0.0 c | 7.88±1.0 a | 7.04±0.1c |
|  | **LSD_0.05_** | 0.11 | 0.09 | 0.08 | 0.09 | 0.11 | 0.13 |
| Foliar spray (mg.L -1) | **D W** | 6.69±0.0 c | 6.41±0.0 d | 6.28±0.0 d | 6.15±0.0 d | 6.84±0.1 c | 6.97±0.1 d |
|  | **SWE 1000** | 6.92±0.0 b | 6.6±0.1 c | 6.45±0.1 c | 6.3±0.0 c | 7.09±0.1 b | 7.25±0.1 c |
|  | **SWE 2000** | 6.91±0.0 b | 6.59±0.1 c | 6.44±0.1 c | 6.3±0.0 c | 7.09±0.1 b | 7.24±0.1 c |
|  | **ADY 3000** | 7.07±0.1 ab | 6.72±0.2 b | 6.56±0.1 b | 6.4±0.1 b | 7.27±0.2 b | 7.43±0.2 b |
|  | **ADY 4000** | 7.26±0.1 a | 6.88±0.3 a | 6.69±0.2 a | 6.51±0.1 a | 9.15±1.4 a | 7.67±0.3 a |
|  | **LSD_0.05_** | 0.23 | 0.12 | 0.08 | 0.09 | 1.13 | 0.14 |

- Means (±SE) within a column followed by the same letter within the same treatment group are **not significantly different** according to the LSD (p ≤ 0.05).
- The data presented in this table are **marginal means**, where values for Light Intensity are averaged across all foliar spray treatments, and values for Foliar Spray are averaged across all light intensity conditions. **For the specific interaction effects (Light × Foliar Spray) and the performance of each treatment combination, please refer to** figure S1
- DW = distilled water; SWE = seaweed extract; ADY=active dry yeast.

**Table S3** Effects of light intensity and foliar sprays (seaweed extract, and active dry yeast) on the stalk length (cm) of rose plants during the 2024 season. Two factor RCBD: factor A = light (full sun, 65%, 75% shade), factor B = foliar spray (DW, SWE1000, SWE2000, ADY3000, ADY4000). Main effect means are marginal means over the other factor.

|  |  | **Stalk length (cm)** | | | | | |
| --- | --- | --- | --- | --- | --- | --- | --- |
| Treatments | **Month** | **May** | **June** | **July** | **August** | **September** | **October** |
| Light Intensity | **Full sun** | 61.24±0.7 a | 60.97±0.7 a | 60.71±0.8 a | 62.45±0.6 a | 62.98±0.8 a | 64.01±0.7 a |
|  | **65%** | 59.86±1.0 b | 59.05±0.9 b | 59.53±0.5 b | 61.19±1.0 b | 61.47±0.8 b | 62.63±1.0 b |
|  | **75%** | 57.85±0.5 c | 57.66±0.6 c | 58.22±0.5 c | 59.18±0.5 c | 59.71±0.6 c | 60.62±0.5 c |
|  | **LSD_0.05_** | 0.72 | 0.56 | 0.73 | 0.71 | 0.79 | 0.75 |
| Foliar spray (mg.L ^-1^) | **D W** | 57.99±0.9 d | 57.6±0.9 d | 58.31±0.7c | 59.32±0.9 d | 59.78±1.0 d | 60.76±0.9 d |
|  | **SWE 1000** | 58.78±0.9 c | 58.29±0.9 c | 58.51±0.4c | 60.11±0.9 c | 60.57±0.8 c | 61.55±0.9 c |
|  | **SWE 2000** | 58.84±0.9 c | 58.62±1.0 c | 58.93±0.8 c | 60.17±0.9 c | 60.5±0.7 c | 61.61±0.9 c |
|  | **ADY 3000** | 60.78±1.1 b | 60.32±0.9 b | 60.43±0.8 b | 62.11±1.1 b | 62.51±1.1 b | 63.55±1.1 b |
|  | **ADY 4000** | 61.87±1.1 a | 61.29±1.1 a | 61.25±1.0 a | 63±1.1 a | 63.59±1.1 a | 64.64±1.1 a |
|  | **LSD_0.05_** | 0.5 | 0.36 | 0.64 | 0.44 | 0.52 | 0.57 |

- Means (±SE) within a column followed by the same letter within the same treatment group are **not significantly different** according to the LSD (p ≤ 0.05).
- The data presented in this table are **marginal means**, where values for Light Intensity are averaged across all foliar spray treatments, and values for Foliar Spray are averaged across all light intensity conditions. **For the specific interaction effects (Light × Foliar Spray) and the performance of each treatment combination, please refer to** figure S2
- DW = distilled water; SWE = seaweed extract; ADY=active dry yeast.

**Table S4** Effects of light intensity and foliar sprays (seaweed extract, and active dry yeast) on the stalk thickness (mm) of rose plants during the 2024 season. Two factor RCBD: factor A = light (full sun, 65%, 75% shade), factor B = foliar spray (DW, SWE1000, SWE2000, ADY3000, ADY4000). Main effect means are marginal means over the other factor.

|  |  | **Stalk Thickness (mm)** | | | | | |
| --- | --- | --- | --- | --- | --- | --- | --- |
| Treatments | **Month** | **May** | **June** | **July** | **August** | **September** | **October** |
| Light Intensity | **Full sun** | 6.07±0.4 a | 6.22±0.2 a | 6.39±0.3 a | 5.65±0.1 a | 5.62±0.1 b | 5.68±0.3 b |
|  | **65%** | 5.51±0.2 c | 6.15±0.1 a | 5.82±0.1 b | 5.62±0.1 a | 5.39±0.1 c | 5.9±0.2 a |
|  | **75%** | 5.85±0.1 b | 6.21±0.1 a | 6.38±0.1 a | 6.01±0.1 a | 5.94±0.1 a | 5.65±0.1 b |
|  | **LSD_0.05_** | 0.49 | NS | 0.359 | NS | 0.31 | 0.43 |
| Foliar spray (mg.L ^-1^) | **D W** | 5.28±0.5 c | 5.95±0.2 c | 5.73±0.3 c | 5.79±0.1 a | 5.43±0.1 b | 5.17±0.3 d |
|  | **SWE 1000** | 5.71±0.3 b | 6.02±0.0 c | 6.2±0.5 b | 5.63±0.2 a | 5.53±0.3 b | 5.63±0.1 c |
|  | **SWE 2000** | 5.73±0.4 b | 6.24±0.2 bc | 6.36±0.3 ab | 5.85±0.1 a | 5.9±0.1 a | 5.87±0.2 bc |
|  | **ADY 3000** | 6.07±0.4 a | 6.26±0.2 ab | 6.23±0.2 ab | 5.73±0.4 a | 5.68±0.2 ab | 6.15±0.1 a |
|  | **ADY 4000** | 6.24±0.3 a | 6.49±0.3 a | 6.47±0.1 a | 5.8±0.1 a | 5.7±0.1 ab | 5.9±0.3 b |
|  | **LSD_0.05_** | 0.24 | 0.24 | 0.25 | 0.26 | 0.39 | 0.25 |

- Means (±SE) within a column followed by the same letter within the same treatment group are **not significantly different** according to the LSD (p ≤ 0.05).
- The data presented in this table are **marginal means**, where values for Light Intensity are averaged across all foliar spray treatments, and values for Foliar Spray are averaged across all light intensity conditions. **For the specific interaction effects (Light × Foliar Spray) and the performance of each treatment combination, please refer to** figure S2
- DW = distilled water; SWE = seaweed extract; ADY=active dry yeast.

**Table S5:** Interaction effects of light intensity and different concentrations of biostimulants on the vegetative growth, chlorophyll content, and nutrient content of *R. hybrida*. Light (full sun, 65%, 75% shade) and foliar spray (DW, SWE1000, SWE2000, ADY3000, ADY4000) during 2023 season.

| **Light regime** | **Treatments** | **Number of leaves per stalk** | **Leaf area (cm^2^)** | **Leaves fresh weight (g)** | **Leaves Dry weight (g)** | **Chlorophyll content (SPAD values)** | **Nitrogen%** | **Phosphorus %** | **Potassium %** | **Zn (ppm)** | **Cu (ppm)** |
| --- | --- | --- | --- | --- | --- | --- | --- | --- | --- | --- | --- |
| Full Sun | **D W** | 11.92 ± 0.25c | 178.89 ±8.3d | 18.65 ±1.3d | 4.21 ±0.1e | 44.64 ±0.91d | 2.56 ±0.2d | 0.47 ±0.02g | 1.5 ±0.1c | 41.4 ±4.3e | 18.51 ±1.5e |
|  | **SWE 1000** | 12.73 ± 0.51c | 248.76 ±7.9b | 29.21 ±1.1b | 5.46 ±0.1c | 48.11 ±0.85c | 2.76 ±0.1c | 0.58 ±0.04f | 1.65 ±0.1c | 67.59 ±3.2c | 26.63 ±1.2c |
|  | **SWE 2000** | 14.93 ±0.48a | 274.4 ±6.6a | 34.17 ±0.9a | 5.72 ±0.0c | 52.02 ±0.65b | 2.92 ±0.1b | 0.7 ±0.04c | 2.33 ±0.1b | 71.75 ±4.1b | 30.52 ± 0.9b |
|  | **ADY 3000** | 16.32 ±0.61a | 282.65 ±8.34a | 36.73 ±0.81a | 6.27 ±0.1b | 46.17 ±0.9d | 2.98 ±0.2b | 0.75 ±0.02b | 1.47 ±0.0c | 82.3 ±4a | 32.42 ±1.5a |
|  | **ADY 4000** | 17.42 ±0.5a | 295.87 ±5.5a | 36.02 ±1.1a | 6.83 ±0.0a | 55.18 ±0.71a | 3.12 ±0.0a | 0.81 ±0.01a | 2.1 ±0.1b | 89.1 ±3.1a | 35.05 ±1.4a |
| 65% | **D W** | 8.81 ±0.35d | 128.66 ±8.2f | 23.09 ±0.9c | 5.2 ±0.1d | 40.95 ±0.82e | 1.42 ±0.1g | 0.44 ±0.0g | 1.59 ±0.1c | 35.74 ± 3e | 17.79 ±1.1e |
|  | **SWE 1000** | 11.49 ±0.21c | 165.11 ±7.5d | 27.53 ±1.2b | 5.51 ±0.1c | 49.08 ±0.9c | 1.55 ±0.2f | 0.56 ±0.02e | 2.47 ±0.0a | 64.19 ±4.3c | 24.96 ±1.5c |
|  | **SWE 2000** | 12.54 ±0.26c | 254.17 ±4.2b | 27.93 ±1.1b | 5.53 ±0.0c | 48.62 ±0.6c | 1.71 ±0.1f | 0.64 ±0.04d | 2.47 ±0.1a | 70.91 ±4c | 25.21 ±1.1c |
|  | **ADY 3000** | 14.7 ±0.5b | 265.76 ±8.3b | 34.62 ±0.9a | 6.12 ±0.1b | 52.85 ±0.9a | 1.9 ±0.0e | 0.76 ±0.03b | 2.33 ±0.1b | 75.02 ±4.3b | 30.47 ±1.1b |
|  | **ADY 4000** | 14.19 ±0.4b | 277.01 ±7.6a | 33.59 ±1.3a | 6.25 ±0.1b | 50.36 ±0.9b | 2 ±0.2d | 0.83 ±0.01a | 3.04 ±0.0a | 78.44 ±3b | 30.11 ±1.4b |
| 75% | **D W** | 6.21±0.5e | 116.75 ±7.2f | 17.13 ±1.1d | 4.19 ±0.1e | 47.78 ±0.34c | 1.25 ±0.05h | 0.41 ±0.02h | 1.2 ±0.1c | 33.4 ±3.2f | 17.27 ±1.3e |
|  | **SWE 1000** | 8.68 ±0.47d | 152.64 ±8e | 25.36 ±1.3c | 5.24 ±0.1d | 45.03 ±0.85d | 1.3 ±0.1g | 0.4 ±0.04h | 1.67 ±0.1c | 61.32 ±1.5d | 21.76 ±1.2d |
|  | **SWE 2000** | 11.63 ±0.2c | 231.22 ±6.2c | 29.6 ±0.9b | 5.3 ±0.0d | 46.05 ±0.71d | 1.32 ±0.2g | 0.45 ±0.02g | 2.23 ±0.1b | 64.13 ±4.1c | 23.29 ±1.5d |
|  | **ADY 3000** | 12.19 ±0.3c | 240.29 ±6.5c | 29.12 ±0.9b | 5.48 ±0.1c | 48.02 ±0.91c | 1.44 ±0.1f | 0.57 ±0.0e | 2.64 ±0.0a | 74.54 ±2.5b | 25.62 ±1.5c |
|  | **ADY 4000** | 12.83 ±0.3c | 249.36 ±5.7b | 27.52 ±0.6b | 5.39 ±0.0d | 47.91 ±0.81c | 1.63 ±0.1e | 0.6 ±0.0e | 2.42 ±0.1b | 76.2 ±3.4b | 26.42 ±1.5c |
|  | **LSD_0.05_** | 1.45 | 23.59 | 3.92 | 0.27 | 2.81 | 0.14 | 0.04 | 0.52 | 7.12 | 3.32 |

- Means (±SE) within a column followed by the same letter within the same treatment group are **not significantly different** according to the LSD (p ≤ 0.05).
- LSD 0.05 = List significant differences at 0.05 probability.
- This data is illustrated in figure 3, 6 and 7.


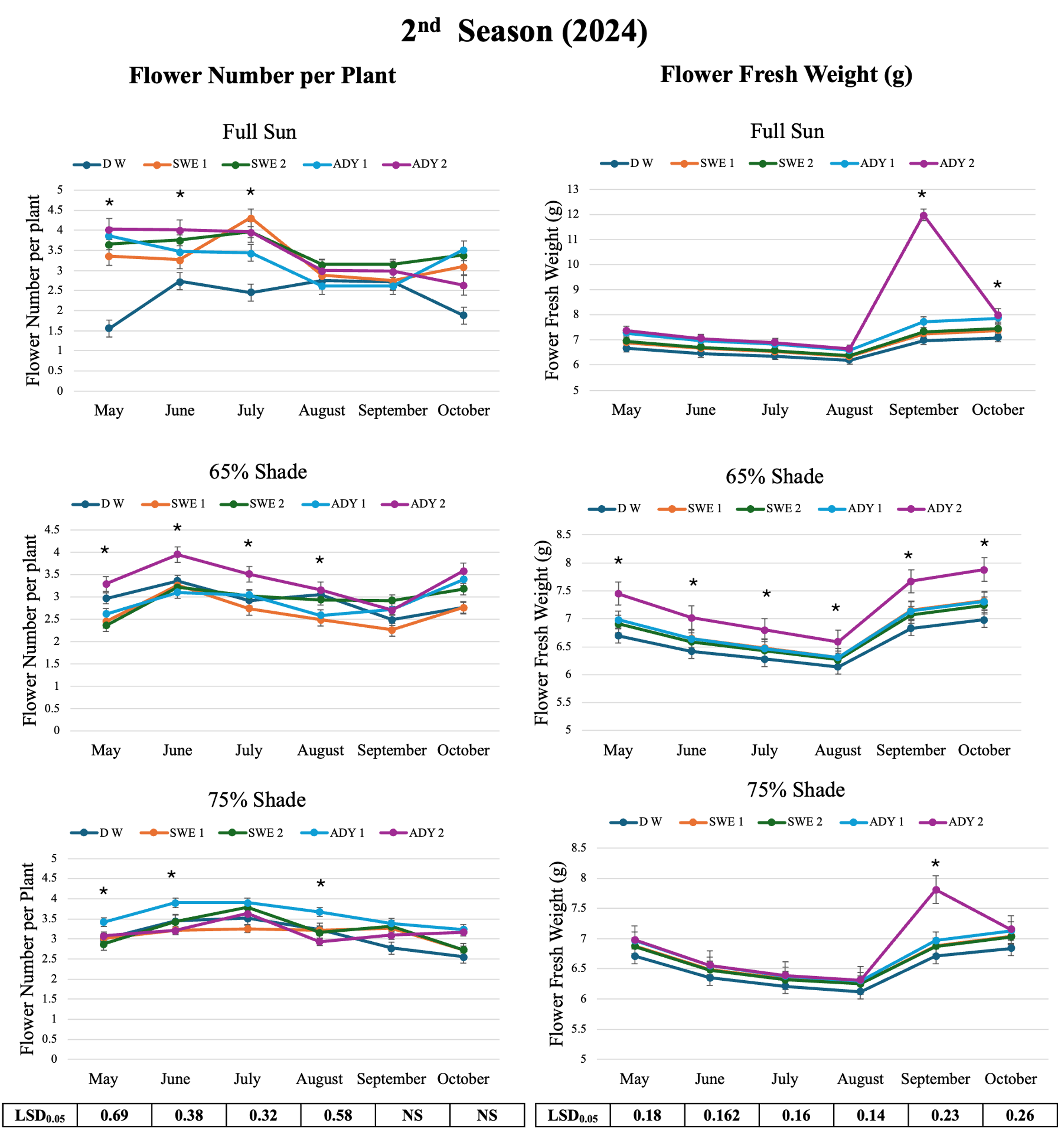


**Fig. S1** Interaction effects of light intensity and different concentrations of biostimulants on the flower productivity of *R. hybrida* during the 2024 season (May-October). LSD = List significant differences at 0.05 probability. Each value represents the mean of five biological replicates (n=5), where one plant was considered as an experimental unit. Means with the asterisk (*) indicates significant differences at p ≤ 0.05 according to Fisher LSD test, within the same light regime, where full sun, 65%, and 75% are the three different light intensities. DW, SWEI, SWE2, ADYI, and ADY2 are the five foliar spray treatments with distilled water, seaweed concentrations of 1000 and 2000, and active dried yeast concentrations of 3000 and 4000 mg L^-1,^ respectively. The error bars represent the standard error (SE).

**
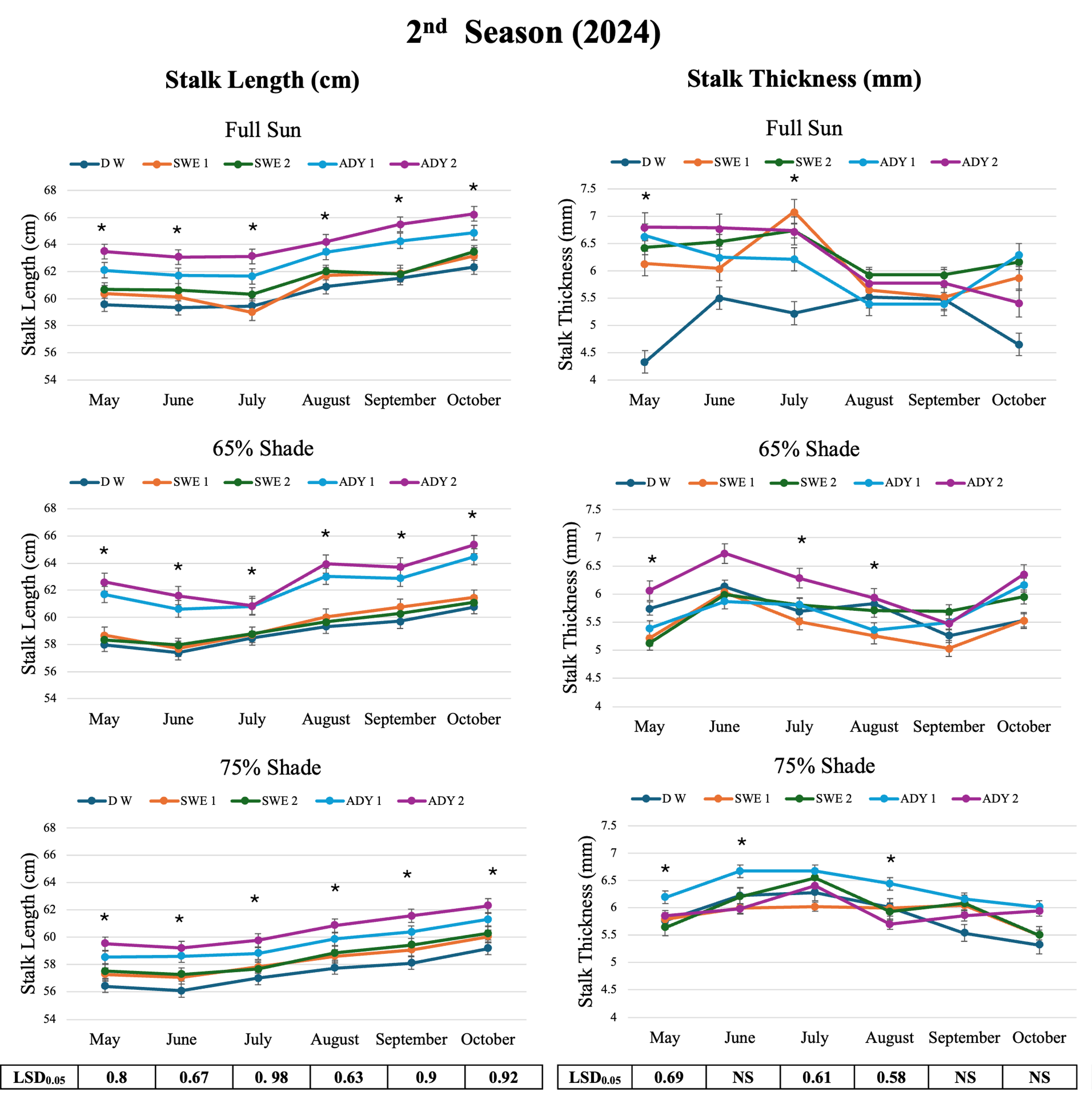
**

**Fig. S2** Interaction effects of light intensity and different concentrations of biostimulants on the stalk quality of *R. hybrida* during the 2024 season (May-October). LSD = List significant differences at 0.05 probability. Each value represents the mean of five biological replicates (n=5), where one plant was considered as an experimental unit. Means with the asterisk (*) indicates significant differences at p ≤ 0.05 according to Fisher LSD test, within the same light regime, where full sun, 65%, and 75% are the three different light intensities. DW, SWEI, SWE2, ADYI, and ADY2 are the five foliar spray treatments with distilled water, seaweed concentrations of 1000 and 2000, and active dried yeast concentrations of 3000 and 4000 mg L^-1,^ respectively. The error bars represent the standard error (SE).
